# Evaluating species identification apps as a tool for small plot-based surveys of vascular plants in Alberta, Canada

**DOI:** 10.1093/aobpla/plag020

**Published:** 2026-05-08

**Authors:** Iyesha M Wanigasinghe, Diane L Haughland, Lysandra A Pyle, Mary A L Villeneuve, Scott E Nielsen

**Affiliations:** Department of Renewable Resources, University of Alberta, 116 St & 85 Ave, Edmonton, Alberta, T6G 2R3, Canada; Department of Renewable Resources, University of Alberta, 116 St & 85 Ave, Edmonton, Alberta, T6G 2R3, Canada; Alberta Biodiversity Monitoring Institute, University of Alberta, 10055 106 St NW, Suite 700, Edmonton, Alberta, T5J 2Y2, Canada; Alberta Biodiversity Monitoring Institute, University of Alberta, 10055 106 St NW, Suite 700, Edmonton, Alberta, T5J 2Y2, Canada; Department of Renewable Resources, University of Alberta, 116 St & 85 Ave, Edmonton, Alberta, T6G 2R3, Canada

**Keywords:** automated species identification, biodiversity monitoring, detectability, flora incognita, identification accuracy, iNaturalist, mobile apps, vascular plants

## Abstract

While mobile apps are becoming more accurate at identifying vascular plants, it is unclear whether accuracy is maintained when doing plot-based surveys, where image acquisition is limited to a small pool of individuals that often lack ideal identification features. We evaluated two free plant identification apps, Flora Incognita and iNaturalist, using 5291 field images of 119 species from 54 plots of 0.8 m^2^, from graminoid-dominated grassland and forested sites. We examined the top-1 identification (ID) accuracy as a function of botanical (growth form and reproductive state) and photographic (image quality and background) parameters. We used score fusion to test whether combining multiple images of the same individual (perspective combination) improved ID accuracy. Top-1 ID accuracy was consistently higher in Flora Incognita (79.2% overall) as compared to 66.5% in iNaturalist. No groups reached the reference expert accuracy of plot-based surveys (90%–95%). Images featuring reproductive structures significantly improved ID accuracy, particularly for graminoids (overall +8.9% in Flora Incognita, +17.1% in iNaturalist), and high-quality images increased accuracy significantly for graminoids and herbs in iNaturalist. Combining multiple images improved the overall ID accuracy of vascular plants in iNaturalist by 6% and 1.7% in Flora Incognita, with significant improvement for herbs and shrubs in iNaturalist. We provide recommendations for ID success in the field for app users as well as app developers to optimize image acquisition for species identification in surveying and monitoring efforts.

## Teaser

While mobile apps are becoming more accurate at identifying vascular plants from photos, it is unclear whether accuracy is maintained when doing small, plot-based surveys, where surveyors are limited to a small pool of often immature or damaged individuals. We tested two mobile apps (iNaturalist and Flora Incognita) in forests and grasslands and found that both performed relatively well (within ca. 10% of the estimated expert accuracy of 90–95%) for herbs, shrubs, and trees. However, graminoid identification accuracy was poor. Accuracy was higher for better quality photos (e.g., in focus and well-lit) and when photos included reproductive structures. Accuracy was also higher when multiple photos of different aspects of the same individual were combined to inform identification, a feature currently available only in Flora Incognita.

## Introduction

Identification of plants in the field requires substantial botanical expertise, particularly in plot-based assessments. In small plot studies (where the plots or quadrats examined typically are ≤1 m^2^), individual plants within the plot may lack key diagnostic features due to immaturity, physical damage (i.e. defoliation), or disease. Shortages in botanists and botanical knowledge, and increasing plant awareness disparity make it increasingly difficult to conduct these studies, even as they become more important due to rapid biodiversity loss (Gaston and O’Neill 2004, Dayrat 2005, Jose *et al*. 2019, Parsley 2020). Automating species identification is a promising approach to mitigate these shortages and reduce the burden of routine identification (Gaston and O’Neill 2004, Bonnet *et al*. 2016, 2018, Jose *et al*. 2019, Pärtel *et al*. 2021).

Increasing computer power, together with recent boosts in data availability, have led to significant advances in AI and machine learning algorithms, notably deep learning and neural network technologies (LeCun *et al*. 2015, Tan *et al*. 2020). Image recognition technology is advancing rapidly, with several automatic plant identification apps freely available for smartphones and tablets (Wäldchen and Mäder 2018a, 2018b, Jones 2020, Truong and Van der Wal 2024). However, the potential of these apps to aid real-world, plot-based field studies remains unexplored. Previous studies evaluating plant identification apps typically focused on subjectively selected individuals or plant groups while avoiding certain species groups (e.g. exotic species) or damaged/infected specimens (Jones 2020, Schmidt *et al*. 2022, Hart *et al*. 2023). A key gap is the lack of assessment of their accuracy under realistic field conditions, where we cannot subjectively select or ignore specimens, as in biodiversity monitoring and floristic survey programmes.

Among the best-performing, freely available apps for species identification are iNaturalist (https://www.inaturalist.org/) and Flora Incognita (https://floraincognita.com/) (Pärtel *et al*. 2021, Hart *et al*. 2023, Rzanny *et al*. 2024). Interestingly, these apps employ different strategies, including computer vision methods, image sourcing, and single versus multiple image analysis. iNaturalist has over 4 million users world-wide and, as of September 2025, over 270 million crowd-sourced observations, of which about 106 million observations (∼40%) are vascular plants (Phylum Tracheophyta) (https://www.inaturalist.org/observations). At the time of our study (2023–2024), the computer vision model of iNaturalist was a convolutional neural network with Xception architecture (François Chollet, unpublished data, https://arxiv.org/pdf/1610.02357; Popp *et al*. 2025). Its computer vision model provides a binary classification indicating whether the user should have confidence in the identification (identification confirmed) or whether multiple potential identifications are suggested in order of likelihood (Hart *et al*. 2023). iNaturalist allows users to submit multiple images for any species observations but analyses each image separately.

Flora Incognita focuses on the central European flora, but is quickly expanding its coverage to North America and beyond (Mäder *et al*. 2021, Popp *et al*. 2025). Image recognition in Flora Incognita uses a cascade of specialized deep convolutional neural network-based computer vision models to process images (Mäder *et al.* 2021, Pärtel *et al*. 2021). The computer vision model is trained exclusively on expert-identified images. Identifications suggested by the classifier need to be confirmed by the user to create a record (Wäldchen *et al*. 2018, Mahecha *et al*. 2021, Mäder *et al*. 2021). Flora Incognita prompts the user to submit multiple images of complementary plant organs to generate a likelihood-based ID based on fusion methods (Mäder *et al*. 2021, Anyomi 2024). This multi-perspective classification approach mirrors how botanists and taxonomists identify specimens by examining different views or structures (i.e. perspectives) to provide complementary information to assign an object into a class, which in this case equals a species (Nguyen *et al*. 2018, Seeland and Mäder 2021). This score fusion method has been shown to increase the accuracy of automated species identification of plants, including graminoids (Joly *et al*. 2014, Nhan *et al*. 2020, Rzanny *et al*. 2022).

Both iNaturalist and Flora Incognita incorporate geolocation data to enhance species identification. iNaturalist uses a dedicated Geomodel that predicts the likelihood of species presence at a given location, and integrates this geo score with visual similarity scores to give a prediction (iNatBlog 2025). In contrast, Flora Incognita employs a multi-model approach that combines image analysis with location embedding (based on presence–absence maps, user occurrence records, soil type, land cover, and phenology, etc.) for predicting likely species at a given location and time (Mäder *et al*. 2021).

Several studies have compared the performance of human experts and plant identification systems using existing online or curated benchmark image datasets with preselected and potentially biased species pools (Goëau *et al*., unpublished data, https://arxiv.org/pdf/2509.21419; Lasseck 2018). While such studies are valuable for controlled and reproducible comparisons of algorithms, acquiring images through fieldwork remains indispensable for capturing how identification systems perform under real-world conditions where species composition, specimen quality, and image quality can vary substantially.

Although many studies have evaluated mobile apps for identifying vascular plants (Jones 2020, Mäder *et al*. 2021, Pärtel *et al*. 2021, Xing *et al*. 2021, Schmidt *et al*. 2022, Hart *et al*. 2023, Anyomi 2024, Jones and Jones 2025, Scholten 2025), most studies are limited to a small sample size; e.g. 38 photos (Jones 2020), <1000 (Schmidt *et al*. 2022, Hart *et al*. 2023, Scholten 2025, but see Popp *et al*. 2025), and/or a limited number of species, reducing their generalizability (Anyomi 2024, Jones and Jones 2025). App evaluations also rarely explore the variation in accuracy attributable to image quality parameters and botanical factors (featured organs, growth form; but see Pärtel *et al*. 2021, Hart *et al*. 2023, Jones and Jones 2025, Popp *et al*. 2025). Another limitation of app-based evaluations is that performance is often assessed using haphazardly or subjectively selected, ideal specimens chosen by the observer in the field, which can lead to inflated accuracy estimates. More systematic evaluation protocols are therefore needed to assess the performance of plot-based vegetation surveys. One such approach was demonstrated by Popp *et al*. (2025), by evaluating app performance by selecting up to nine representative species within standardized plot areas (100 m^2^ in forested habitats and 9 m^2^ in non-forested habitats). However, small-plot studies (≤1 m^2^) that require complete species inventories may pose additional challenges, as ideal specimens may not be available within the limited plot area.

In this study, we address several of these gaps by comparing the accuracy of iNaturalist and Flora Incognita in identifying plants in 0.8 m^2^ plots in two contrasting and common habitat types (forests versus grasslands). Our objectives were two-fold. First, using photo surveys of individual plants in plots, we tested how ID accuracy varied by growth form (tree, shrub, herb, graminoid), reproductive state (reproductive vs. non-reproductive), image quality (low, average, high), and image background (single-species vs. multi-species focused). We hypothesized that accuracy would be lower than in previous studies because of the plot-based limitations identified above. Exploring parameters under the observer’s control (image quality, focus) and those external to the observer (growth form, reproductive state) enabled us to provide recommendations and best practices for using apps in identifying vegetation in plot-based studies. Secondly, we examined how combining multiple perspectives affected identification accuracy for vascular plants, an approach currently employed by only Flora Incognita.

## Methods

### Field work

In 2023, plots were established at three sites in each of the Grasslands Natural Region and the Parkland/Boreal Natural Regions transition in Alberta, Canada (Downing and Pettapiece 2006, Fig. 1, Supplementary Table S1). Grassland sites were located within the Dry Mixedgrass Prairie, each on different grassland soil types (sandy, loamy, and thin eroded soils). This region is semi-arid and graminoid-dominated, typified by mixtures of short to mid-height bunchgrasses and rhizomatous graminoids, receiving most precipitation during early growing season thunderstorms (Downing and Pettapiece 2006, Alberta Parks 2015). During the 2023 field trial, this area received on average 124 mm of precipitation, less than half of the 30-year average from 1992 to 2022 (303 mm) (Alberta Climate Information Service 2024). Plant growth and development were impacted, with fruiting and flowering plants limited. Forested sites occurred on soils with different nutrient and moisture conditions (upland sandy, upland loamy, and riparian) and supported different understory communities. Poplars (*Populus tremuloides* or *Populus balsamifera*) and white spruce (*Picea glauca*) dominated the canopy. The riparian forest site within the municipality of Edmonton supported numerous woody plants not native to central Alberta. Precipitation in central Alberta (406 mm) was marginally below the 30-year average from 1992 to 2022 (429 mm) (Alberta Climate Information Service 2024); however, the growth and development of plants was not notably impacted.

**Figure 1:**
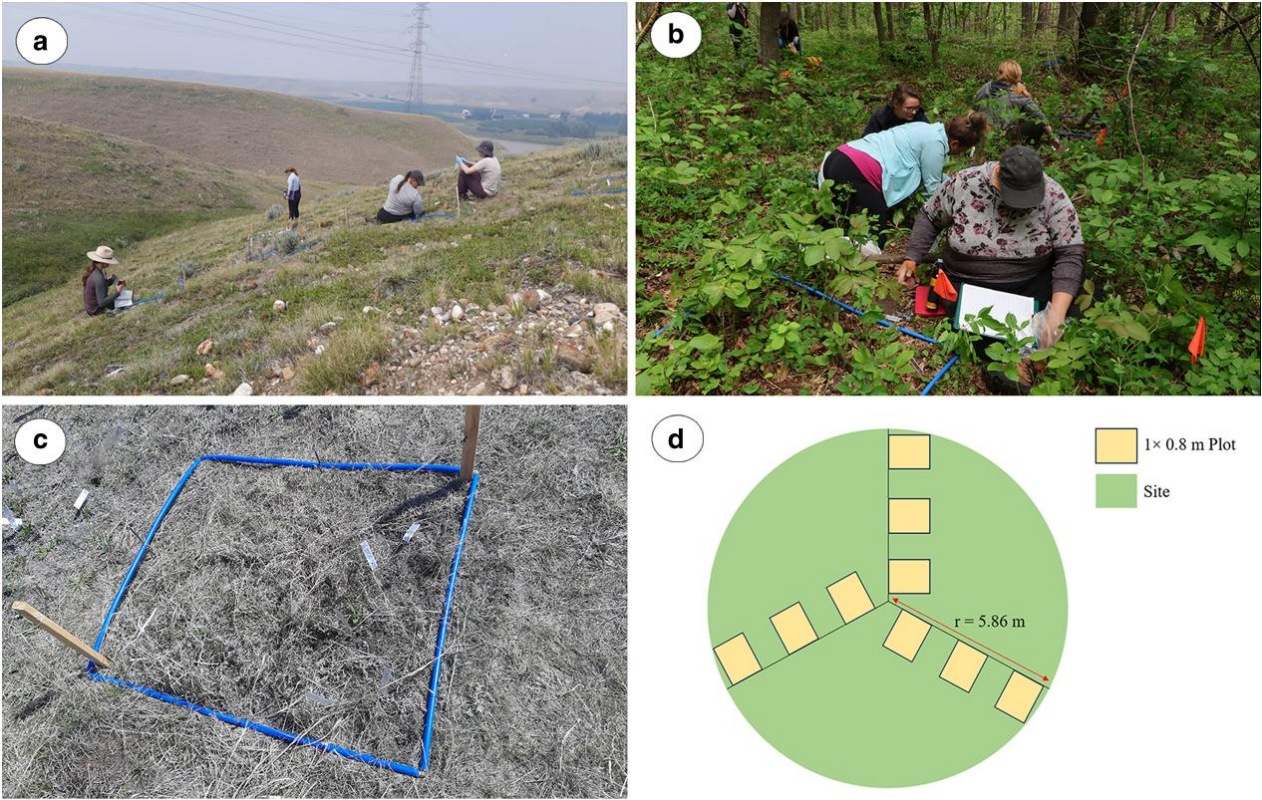
Overview of study design and habitats. (a) Example of a grassland site—MTCL2. (b) Example of a forest site—LFG. (c) 1 × 0.8 m quadrat within a grassland site in early May. (d) 100 m^2^ experimental site design.

At each site, we established nine 1 × 0.8 m plots (Fig. 1), with plot size selected to facilitate plot-level photography. Sites were surveyed using traditional morphology-based methods by two experts (LAP and MALV) for vascular plants. Concurrently, another team photographed at least one individual per species per plot, adding individuals as time allowed. The experts marked individuals to guide photography. By marking individuals, we controlled in-field plant selection to ensure that both experts and AI evaluated the same specimens, thereby eliminating any difference in results due to evaluating different individuals. Field work was conducted in the spring (May 2023) and repeated in the anticipated peak bloom period (July 2023) to capture ephemeral species and different stages of fruiting and flowering for vascular plants. Weather and lighting conditions varied during surveys, but no surveys were conducted in the rain. Both photo and expert surveys were time-unlimited. We used the time of the first and last photo to estimate photo survey time. The time required for expert surveys was not recorded in the field; instead, experts estimated their survey time based on field experience. Photo surveys took an estimated 69 ± 37 SD minutes per plot (minimum 8–maximum 175 minutes; we required each individual plant to be photographed multiple times—see below), whereas expert surveys were estimated at 10–20 minutes per plot.

### Image acquisition and coding

**Figure 2:**
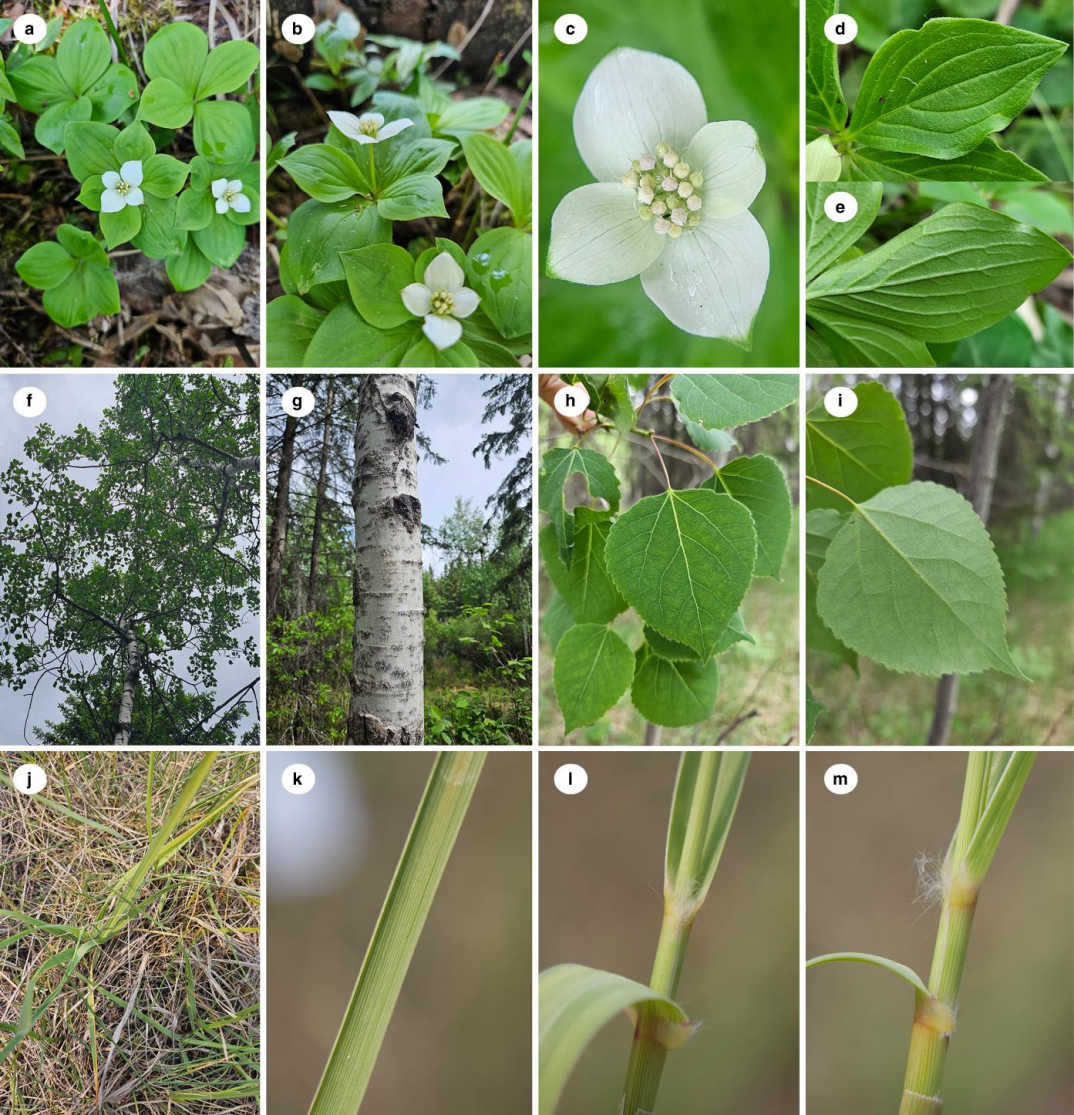
Example image perspectives of vascular plants. (a–e) *Cornus canadensis*: entire plant above (a); entire plant side (b); flower frontal (c); leaf top (d); leaf back (e). (f–i) *Populus tremuloides*: entire plant below (f); stem (g); leaf top (h); leaf back (i). (j–m) *Sporobolus rigidus*: entire plant above (j); leaf top (k); ligule front (l); ligule side (m).

To evaluate the importance of combining multiple perspectives for identification accuracy (Fig. 2), we photographed individuals multiple times, focusing on different views/organs at decreasing focal distance (Supplementary Table S2). The distance between the camera and the plants was reduced until the entire organ or plant was in focus. Overall, photographs were generated by three individuals using a range of smartphone models (iOS + Android). External macro lenses (Moment macro lens M-series 10×, ×10 Apexel, HB100 mm) were used where appropriate to capture more detail. All the images were captured in JPG format, and the location tag was enabled in the settings of the cameras to collect geolocation data. All the images from the cameras were taken under default settings without the application of any effects or filters.

We collected at least one voucher specimen of each species, and additional vouchers when needed to verify their identification in the laboratory. Field identification of approximately 11% of total observations recorded by experts were refined in the laboratory, of which the majority of species were herbs (*Pyrola asarifolia, Boechera retrofracta*, etc.) and graminoids (*Carex duriuscula, Calamagrostis canadensis, Pascopyrum smithii*, etc.). Additional image metadata, including taxa-specific tags (Supplementary Table S3), were embedded into the existing image metadata of each image using Adobe Bridge 2023 (version 14.0). All images were verified by LAP. After tagging, the image metadata was extracted to a tabular format using ExifTool (version 12.62, https://exiftool.org/). The taxonomic authority used for naming vascular plants was VASCAN (https://data.canadensys.net/vascan/search). Image quality was assessed and coded as low, average, or high based on the clarity, focus, lighting, and exposure (Supplementary Table S2, Supplementary Figure S1). Images were coded as single species focused (a single target species was in focus) or as multiple species focused.

### Image testing

Images were bulk-uploaded to a secure server of Flora Incognita in March 2024. All species (119) of vascular plants (5291 images) encountered were uploaded despite Flora Incognita not being trained on seven species common to Alberta, so that we could assess how the convolutional neural network (CNN) model assigns species identifications and confidence estimates for novel species. For each submitted image, the Flora Incognita CNN provided the top 10 identifications and their respective confidence percentages. The results were converted from JSON to a CSV using R version 4.2.3 (R Core Team 2023); packages: *jsonlite* (https://cran.r-project.org/web/packages/jsonlite/index.html), *tidyverse* (Wickham *et al*. 2019). Images were submitted in batches for testing using the iNaturalist Application Programming Interface; RapidAPI-VisionAPI (v1) in August 2024, implemented in R (version 4.2.3) with the packages *httr* (https://cran.r-project.org/web/packages/httr/index.html) and *purr* (https://cran.r-project.org/web/packages/purrr/index.html). Images with large file sizes were resized and compressed to keep the target file size under 3800 kb while not reducing the image quality by more than 95%. For API requests, VisionAPI does not read the location data automatically from images, so we extracted geolocation data manually and submitted it within the R code when making API requests. For each image submitted, identification (up to 10 identifications) and respective confidence percentages were provided by iNaturalist. Results from both apps were combined with *a priori* metadata coding and in-field expert identifications for statistical analyses of species identification success.

### Taxonomy and taxonomic grouping

Nomenclature from mobile applications and expert identifications were reviewed and revised to a consistent taxonomic standard, ensuring that synonyms were not incorrectly coded as misidentifications. We used the following databases: Alberta Conservation Information Management System (ACIMS 2022), Database of Vascular Plants of Canada (VASCAN), Catalogue of Life, Global Biodiversity Information Facility-Application Program Interface Reference, NatureServe, and Alberta Biodiversity Monitoring Institute (ABMI) Taxonomic Workbench (an unpublished online tool for ABMI taxonomists to verify and update taxonomy, which reflects ACIMS).

For all highest-probability identifications from the apps and expert identifications, we grouped species that we did not think could be identified from field images alone (Supplementary Table S4). Visual taxonomic groupings were based on expert knowledge of the group and research using the aforementioned resources. Examples of groupings include geographic species with minor morphological differences (e.g. *Campanula rotundifolia* complex), or with distinguishing features rarely captured in field photography (e.g. minute difference in leaf surfaces, such as the *Solidago canadensis* complex), and local plants indistinguishable without reproductive parts even to experts (e.g. *Thalictrum venulosum*, *T. sparsiflorum*, *T. occidentale*). We did not lump species that were visually similar, but which occurred in different habitats and thus should have different environmental cues that AI could use to distinguish them. Both AI and expert identifications were converted to these taxonomic groups as needed, and the resulting data were used to assess identification accuracy.

### ID accuracy

For each image, the identification with the highest confidence score (top-1 suggestion) from each app was used to assess accuracy, assuming that field technicians would be ill-equipped to choose a lower probability identification. Both apps typically provide an identification only when they reach a certain level of confidence, and they refrain from suggesting an ID when the confidence is below a specific threshold (Jones 2020, Hart *et al*. 2023). For example, in Flora Incognita, if the best suggestion has a score below 0.2, the app will not return a suggestion. If the score is between 0.2 and 0.7, it will return more than one suggestion (Wäldchen, J., personal communication; 2 July 2025). To more accurately interpret our results in the context of how the app returns IDs during field use, we excluded 209 images from the ID accuracy analysis as the top-1 suggestion for those observations had confidence scores below 0.2. In iNaturalist, any threshold considerations are applied before a response is returned via the API, similar to the mobile app (Shepard, A., personal communication; 1 July 2025). Therefore, we analysed the entire dataset without excluding any observations.

To put our results in context, we derived benchmarks from the literature on mobile app accuracy studies and from estimates of accuracy for traditional, plot-based expert plant surveys. We reviewed 14 published studies between 2019 and 2025 that evaluated mobile apps for vascular plant identification and calculated the average reported accuracy across these studies to serve as a reference point. Reported accuracy ranged between 44% and 98.8%, with an average of 73.5% ± 14.7% SD. We used 73.5% as our benchmark, acknowledging variation across studies in the apps evaluated, species tested, study regions, and the number, and quality of images tested (Supplementary Table S5). We derived a range of traditional expert survey accuracy (90%–95%) from Morrison (2016), based on his review of 59 studies that quantified observer errors in vegetation surveys. Morrison (2016) reported mean pseudo-turnover rates ranging from 10% to 30% and misidentification rates of 5%–10%.

### Multiple-perspective combination via score fusion

The purpose of multiple-perspective combination is to leverage the confidence scores of multiple images to improve identification performance. To combine the predictions for multiple perspectives, we adopted the score-level fusion method of Flora Incognita, based on a simple, normalized sum rule, i.e. a summation of multiple scores to provide a single fused score (Nhan *et al*. 2018, Rzanny *et al*. 2019). The fused score *S* over the set of *P* of selected perspectives *p* ∈ *P* is calculated as the sum of the individual scores *s_p_*:


$$S\;=\;\underset{\;p\epsilon P}{\sum }\frac{s_{p}}{\left|P\right|}\;$$

We used the sum rule for its robustness to score variation and lower sensitivity to noisy data, extreme values, and uncertainties in individual output compared to other fusion methods like the product rule and max rule (Nhan *et al*. 2018, 2020). Moreover, this is the most comprehensible method and allows a straightforward interpretation of the results (Rzanny *et al*. 2019, Rzanny *et al*. 2022). The number of perspectives combined for a fused score varied between 2 and 8 (median = 4) in both apps.

### Statistical analysis

We compared species-level top-1 accuracy between two apps (measured as the proportion of images identified accurately for each species), using the Wilcoxon signed-rank test (non-parametric alternative to the paired *t*-test). We used chi-square tests to examine overall associations between identification success and each image covariate. We conducted a three-way stratified Mantel–Haenszel Chi-square analysis for both apps to assess whether top-1 ID success (correct vs. incorrect) is associated with image covariates (reproductive state, image quality, and image background) across growth forms. We excluded trees from the three-way analysis because of low sample size. We did *post hoc* chi-square tests within each growth form to explore within-group associations between image covariates and identification success. Effect sizes for *post hoc* results were calculated using Cramer’s *V* to quantify the strength of association, interpreted as small (≥0.1), medium (≥0.3), or large (≥0.5) effects (Warmbrod 2001, Cetinkaya 2025). For both apps, we assessed the impact of score fusion using Wilcoxon signed-rank tests (species-level ID accuracy as measured by the proportion of images identified accurately versus the proportion of individuals identified accurately after score fusion) and chi-square tests (proportion of individuals identified accurately after applying the score fusion method across growth forms).

## Results

### Overall app accuracy

**Figure 3:**
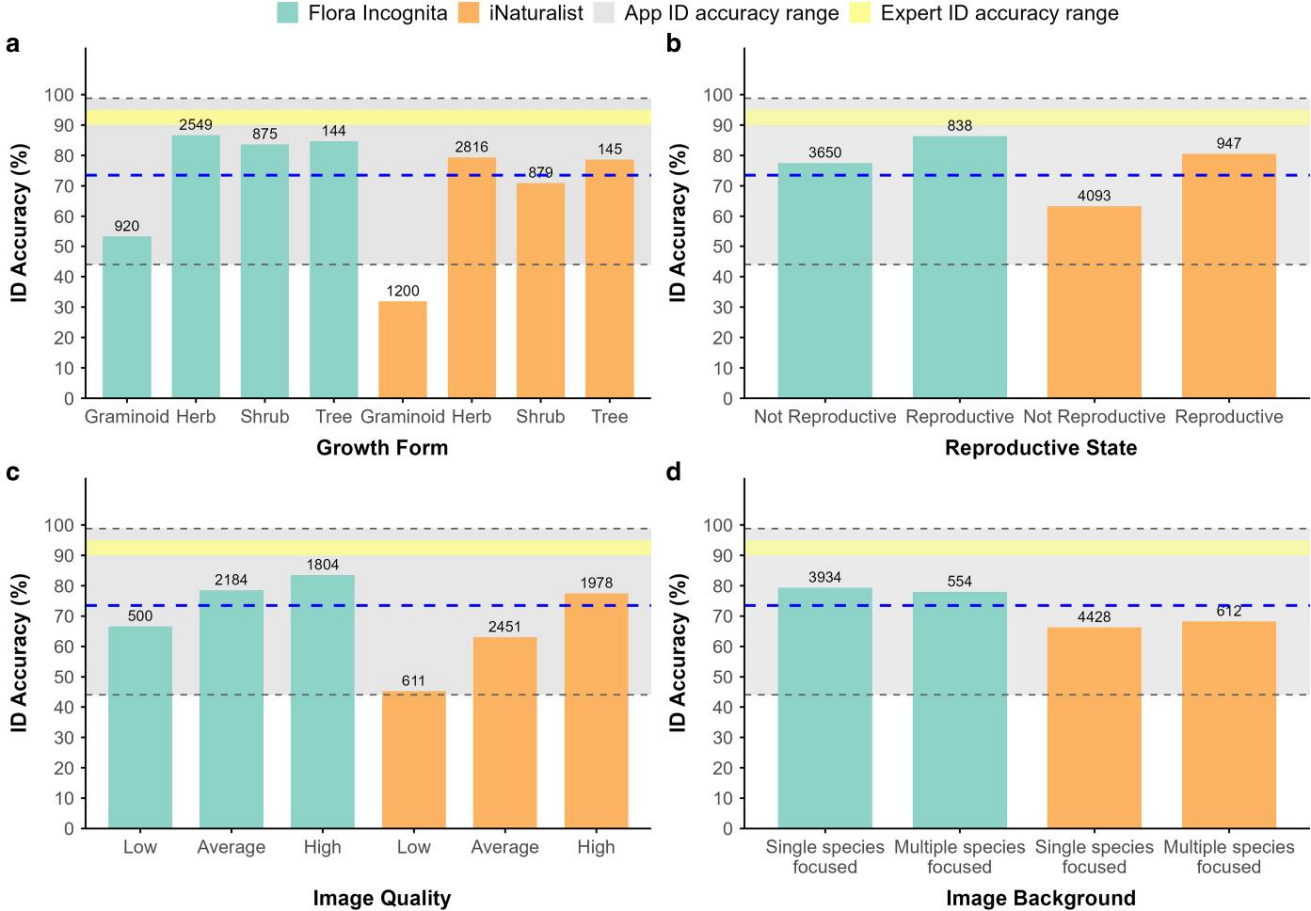
Overall ID accuracy across growth forms and image covariates: (a) growth forms; (b) reproductive state; (c) image quality; (d) image background. Reference values: for both apps, the grey shaded area is the range of mobile app ID accuracy from the literature; grey dashed lines indicate the minimum and maximum app recorded ID accuracy; the blue dashed line indicates the average app recorded ID accuracy; the yellow shaded area represents the reference ID accuracy range for plot-based expert field surveys.

Species-level accuracy was higher in Flora Incognita than in iNaturalist (Fig. 3, Wilcoxon signed-rank test *V* = 3719.5, *P* < .001). For Flora Incognita, accuracy was 79.2% for species and 88.4% for genera, and did not reach the range of expert accuracy (determined by Morrison 2016). Of 112 species, 25 (22%) were identified 100% accurately, and the accuracy of 74 species (66%) exceeded the mean reference app accuracy of 73.5%. Species below the mean reference accuracy were predominantly graminoids. Of the 7 species not trained in Flora Incognita, 61.5% of images were correctly identified to genus (Supplementary Table S7). In iNaturalist, accuracy was 66.5% for species and 73.7% for genera, and was below the range of expert accuracy. Of 119 species of vascular plants tested, 10 species (8%) were identified 100% accurately, and the accuracy of 59 species (50%) exceeded the mean reference app accuracy (Supplementary Table S6–S8).

### Effect of botanical and image parameters

All parameters significantly affected accuracy except image background (single vs. multiple species focused, Table 1). The largest effect was whether a plant was a graminoid or not; all other growth forms (Supplementary Table S9) had similarly high ID accuracy (Fig. 3). In both apps, all growth forms, except graminoids, fell within the range of reference ID accuracy of mobile apps (Fig. 3a). Images depicting reproductive features and high-quality images had the highest accuracy in both apps (Table 1, Fig. 3b and c). Images of herbs and graminoids with reproductive structures were significantly more often identified accurately by both apps (*P* < .001, Table 1). Reproductive structures had the largest positive impact on graminoid identification accuracy (Table 1). High-quality images were significantly more often identified accurately across all growth forms in iNaturalist but only for herbs in Flora Incognita. Overall, image background (single-species vs. multiple-species focused) had no effect on ID success for either app (Fig. 3d). However, for graminoids, single-species focused images were significantly more often identified accurately in Flora Incognita (Table 1).

**Table 1:** Effect of covariates on photo ID accuracy as estimated via three-way stratified Mantel–Haenszel chi-square analysis and post-hoc chi-square tests for Flora Incognita and iNaturalist.

		Flora incognita					iNaturalist				
		*χ* ^2^	*df*	*P*	ID success	Cramer’s *V* (Effect size)	*χ* ^2^	*df*	*P*-value	ID success	Cramer’s *V*
Growth form	Overall	475.8	3	**<**.**001**	Herb > Tree > Shrub > Graminoid		870.1	3	**<.001**	Herb > Tree > Shrub > Graminoid	
Reprod. state × Growth form	Overall	85.6	1	**<**.**001**			170.5	1	**<**.**001**		
	Graminoid	81.8	1	**<**.**001**	Reprod. > Non-reprod.	0.3	253.9	1	**<**.**001**	Reprod. > Non-reprod.	0.5
	Herb	25.0	1	**<**.**001**	Reprod. > Non-reprod.	0.1	28.2	1	**<**.**001**	Reprod. > Non-reprod.	0.1
	Shrub	1.4	1	.23	No effect	0	0.5	1	.479	No effect	0
Image quality × Growth form	Overall	18.4	2	**<**.**001**			100.6	2	**<**.**001**		
	Graminoid	5.8	2	.110	No effect	0.1	31.6	2	**<**.**001**	High > Average > Low	0.2
	Herb	17.0	2	.**001**	High = Average > Low	0.1	73.5	2	**<**.**001**	High > Average > Low	0.2
	Shrub	0.1	2	.962	No effect	0	6.2	2	.**046**	High > Average > Low	0.1
Image background × Growth form	Overall	1.7	1	.187	No effect		0.2	1	.660	No effect	
	Graminoid	11.8	1	.**002**	SF > MF	0.1	3.6	1	.169	No effect	0.1
	Herb	0.6	1	.905	No effect	0	1.1	1	.574	No effect	0
	Shrub	0.0	1	1.000	No effect	0	0.6	1	.574	No effect	0

Reprod. = Reproductive, *χ*^2^ = chi-square statistic, *df* = degrees of freedom, Cramer’s *V* = effect sizes for post-hoc tests; small (≥0.1), medium (≥0.3), or large (≥0.5) effects. Significant results are bolded.

### Multiple perspective combination

**Figure 4:**
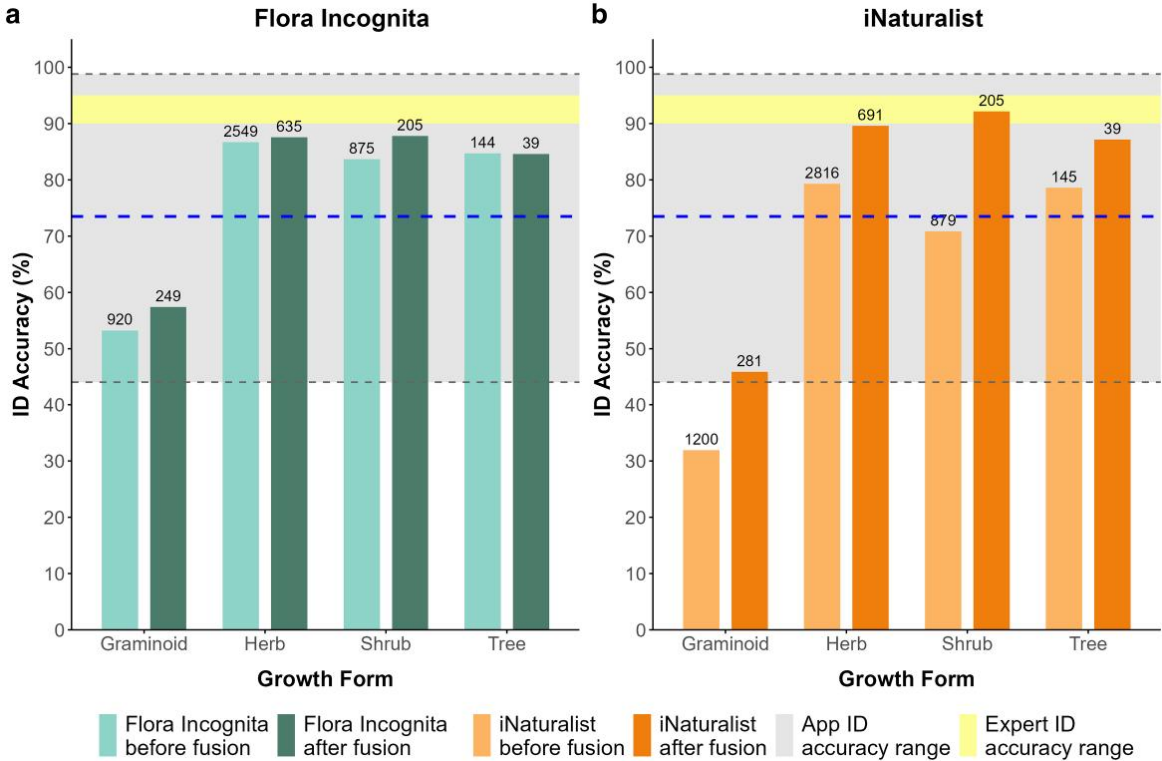
Comparison of species-level ID accuracy by growth form before and after applying score-fusion in (a) Flora Incognita and (b) iNaturalist. Numbers above bars before score fusion equal the number of photos, and after score fusion equal the number of individuals.

Overall ID success was improved by score fusion in both apps (Wilcoxon signed rank test for species-level ID accuracy before and after score fusion, iNaturalist: *V* = 815.5, *P* < .001, Flora Incognita: *V* = 924.5, *P* = .002, Fig. 4). In Flora Incognita, the overall ID accuracy was 80.9% at the species level, a 1.7% improvement. However, the overall accuracy did not improve at the genus level. The effect was larger in iNaturalist: species-level ID accuracy improved to 72.5% (6% improvement) and genus-level to 79.9% (6.2% improvement). ID accuracy improved for 56 species (50% of total species) in Flora Incognita and 82 species (69% of total species) in iNaturalist (Supplementary Table S12). It decreased ID accuracy for 22 species in Flora Incognita and 19 species in iNaturalist. For seven species (of which four were graminoids: *Bromus ciliatus*, *Carex inops*, *Juncus balticus*, and *Pascopyrum smithii*), perspective combination reduced accuracy in both apps. Improvement in ID accuracy varied across different growth forms in both apps (Fig. 4). In iNaturalist, ID success improved significantly for herbs (*P* = .039) and shrubs (*P* < .001) after score fusion. However, the effect sizes were small (Table 2).

**Table 2:** ID accuracy by growth form before and after applying perspective combination (*χ*^2^ = chi-square statistic, *df* = degrees of freedom, Cramer’s *V* = effect sizes for post-hoc tests; small (≥0.1), medium (≥0.3), or large (≥0.5) effects).

App	Growth form	ID accuracy (%)				Chi-square analysis			
		Before score fusion (photos)	After score fusion (individuals)	ID accuracy change	Direction of change	*χ* ^2^	*df*	*P*-value	Cramer’s *V*
Flora Incognita	Graminoid	53.3	57.4	4.2	↑	1.2	1	.272	0.03
	Herb	86.7	87.6	0.9	↑	0.3	1	.612	0.01
	Shrub	83.7	87.8	4.1	↑	1.9	1	.171	0.04
	Tree	84.7	84.6	0.1	↓	0.0	1	1.000	0.00
iNaturalist	Graminoid	31.9	45.9	14.0	↑	2.1	1	.146	0.04
	Herb	79.3	89.6	10.3	↑	4.3	1	.**039**	0.04
	Shrub	70.9	92.2	21.3	↑	13.8	1	**<**.**001**	**0**.**12**
	Tree	78.6	87.2	8.6	↑	0.4	1	.546	0.06

Significant results are bolded, direction of accuracy change indicated with arrows: ↑—increased, ↓—decreased. Sample sizes are indicated in Fig. 2.

**Figure 5:**
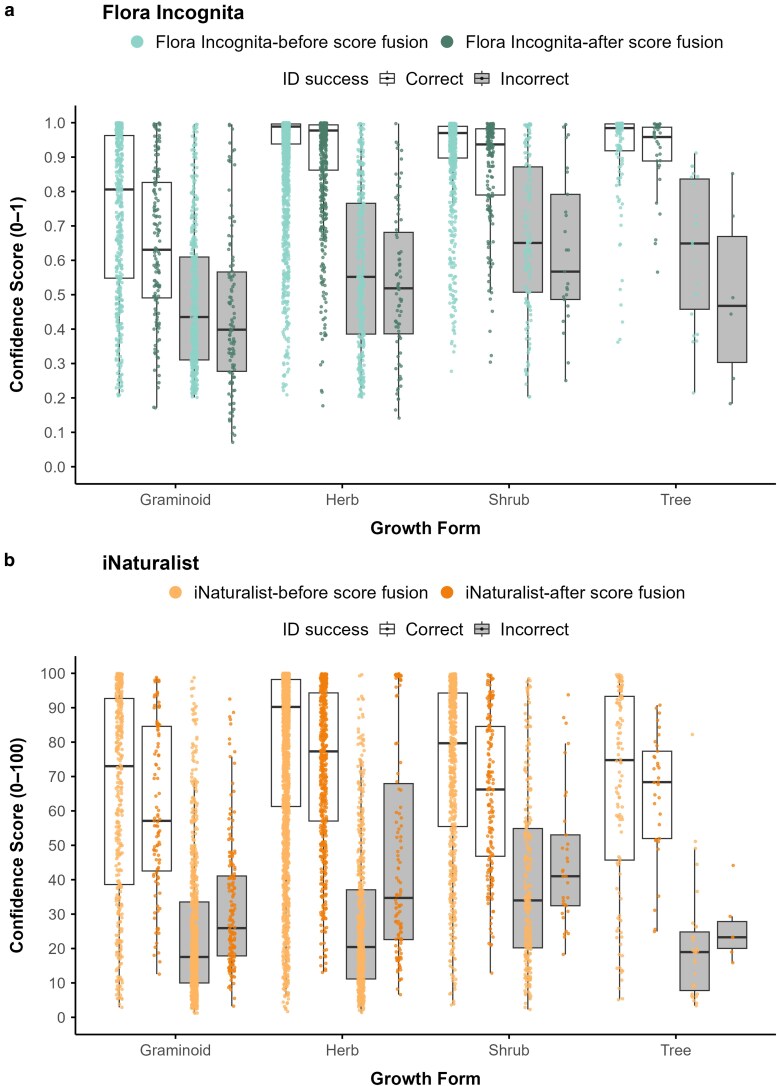
App confidence scores by growth form before (photos) and after (individuals) score fusion, from (a) Flora Incognita (on a 0–1 scale) and (b) iNaturalist (on a 0–100 scale). Score values before score fusion were provided by the apps, and for Flora Incognita, were filtered to scores ≥ 0.2; score values after fusion were calculated herein and not filtered. Boxplots show the median, interquartile range (25th–75th percentile), and whiskers extending to 1.5× the interquartile range. Boxes represent confidence scores in correct (white) and incorrect (grey) identifications. Individual points represent observations. Sample sizes are indicated in Fig. 4.

For both apps, incorrect suggestions were made with low confidence compared to correct suggestions (Fig. 5). Overall, iNaturalist suggestions were made with lower confidence in both its correct and incorrect identifications compared to Flora Incognita. In both apps, confidence scores for correct identifications were highest for herbs and lowest for graminoids, both before and after score fusion (Supplementary Table S10). Score fusion generally diminished confidence scores despite increasing species-level ID accuracy (Fig. 5).

## Discussion

App accuracy did not meet the minimum estimated expert identification accuracy (90%) derived from Morrison (2016) for most species in plot-based surveys. However, 45% (Flora Incognita) and 23% (iNaturalist) of the total species surpassed 90% top-1 accuracy of individual photos. Focusing on reproductive features and high-quality photos generally increased identification accuracy. Graminoids were the exception, with accuracy in both apps below the mean accuracy reported in other studies, as well as estimated expert field accuracy. Graminoid ID accuracy also improved with single-species focused imagery, a factor that other growth forms were robust to. Employing apps, at least as done herein, also required more field time; taking multiple photos of each individual species required two to three times as much field time as expert botanist visual surveys. Surprisingly, we found that the Europe-based Flora Incognita achieved higher accuracy across all growth forms and image covariate comparisons than iNaturalist. Flora Incognita accuracy approached botanical expertise for herbs, shrubs, and trees (Flora Incognita 83.7–86.7% vs. 90%–95% estimated expert accuracy). Regardless, a finding common to both apps was the value of score fusion across multiple images/perspectives. Score fusion significantly increased app accuracy, with the greatest gains observed for iNaturalist.

The lower ID accuracy in iNaturalist is consistent with other studies (Anyomi 2024, Scholten 2025). The computer vision model for iNaturalist was trained on 90 290 taxa (https://www.inaturalist.org/blog/archives/2024/08), compared to only 16 000 taxa in Flora Incognita (Wäldchen, personal communication; 2023). As a general-purpose model trained across diverse life forms, iNaturalist may perform less accurately than Flora Incognita, which is primarily plant-focused. Furthermore, distinguishing among more classes inherently increases the classification difficulty for CNNs (Luo *et al*. 2019, Seeland *et al*. 2019). Moreover, the two apps follow different approaches to train their models with iNaturalist based on crowd-sourced research-grade observations, while in Flora Incognita it is strictly expert-curated. The presence of misidentified records in iNaturalist is well-documented and stems from a lack of expertise among some observers and even prolific identifiers (McMullin and Allen 2022, López-Guillén *et al*. 2024). This results in the use of incorrect ‘research-grade’ observations during model training on iNaturalist. Because the two apps differ in multiple aspects, it is not clear which aspect is lowering iNaturalist’s identification success in this study.

In both apps, differences in reported accuracy across growth forms, species, and depicted plant views/organs may stem from imbalanced training datasets. Mobile apps are trained on imbalanced datasets with skewed frequency of occurrences for classes/species, resulting in variable qualities and quantities of information available for image classification tasks (Joly *et al*. 2014, Xing *et al*. 2021). These data imbalances can stem from sampling imbalance (caused by natural frequency, rarity, and geographical and seasonal sampling bias), content imbalance (related to the focused image content: reproductive vs. non-reproductive features, etc.), or taxonomic imbalance (caused by variation in the number of species grouped into genera and families in the training datasets) (Seeland *et al*. 2019, Scholten 2025).

Observation efforts are often biased towards charismatic species. Cryptic and taxonomically challenging plants with small, inconspicuous flowers (e.g. Poaceae, Cyperaceae, Amaranthaceae, Brassicaceae) are less observed and underrepresented in records, as relatively few observers possess the expertise or interest required to identify them reliably from photographs. Therefore, we can expect varied identification success across different growth forms, image perspectives, and between apps due to imbalanced representation in the training datasets. Finally, field identification of plants relies on the observer’s perception of three-dimensional structures, as well as additional sensory cues such as scent (e.g. volatile organic compounds released from glands or crushed leaves) and tactile feedback (e.g. texture, tissue density), data not available for photo-based identification (Wäldchen *et al*. 2018, Schmidt *et al*. 2022).

Previous studies evaluating identification accuracy across broader taxonomic groups have identified Poaceae to be among the families with the lowest accuracies (Rzanny *et al*. 2019, Pärtel *et al*. 2021), which was true for our study as well. Graminoids, due to their similar visual appearance and inconspicuous reproductive features, represent a major challenge for image-based identification, especially when they are non-flowering (Rzanny *et al*. 2022). Narrow lamina, minute vegetative features like ligules or auricles, and small, often concealed florets, which are important features for identification to the species level, make photographing graminoids challenging in the field, particularly under strong winds and high illumination. In addition, Scholten (2025) found that as the local representation of the genus (i.e. the number of species in a genus that are found within the region of investigation) increases, the general accuracy of mobile apps decreases significantly. We observed this for two genera present in our study, *Carex* and *Solidago*. For example, among the six *Carex* species we observed, four species had < 60% accuracy in both apps, with the lowest accuracy for *C. inops* (11% in Flora Incognita and 5% in iNaturalist, Supplementary Table S8).

In common with previous studies (Pärtel *et al*. 2021, Hart *et al*. 2023, Anyomi 2024), we observed significantly higher identification accuracy in both apps for images depicting reproductive features. This increase was highest for graminoids; we observed 32.5% and 50.1% higher accuracy in Flora Incognita and iNaturalist, respectively, compared to images without reproductive structures. Image quality was the next most important covariate (Hart *et al*. 2023, Munzi *et al*. 2023, López-Guillén *et al*. 2024, but see Jones and Jones 2025), with high-quality images generally resulting in higher ID accuracy. In iNaturalist, high-quality images improved ID accuracy across growth forms 15%–23% as compared to both low and average quality images (Supplementary Table S11). In Flora Incognita, however, both high- and average-quality images across all growth forms yielded similarly high ID accuracy, another benefit with this app, considering how difficult it can be to acquire high-quality images in the field.

Contrary to previous studies (Pärtel *et al*. 2021, Hart *et al*. 2023), we observed only slight differences in overall accuracy (1.4% in Flora Incognita and 2% in iNaturalist) for images focused on the target species versus images with multiple plant species in focus for most growth forms. The exception was graminoids, where we found 19.6% improvement in Flora Incognita and 9.4% in iNaturalist when a single species was in focus. In most mobile apps, where a single classifier is trained on all images irrespective of different perspectives/organs, there could be a lot of confounding background information that enters the visual space of the network for graminoids (Rzanny *et al*. 2017, 2019). Therefore, rather than identifying the target species, they tend to identify another species in the background that the observer did not intend. If an image contains poorly defined edges or cluttered backgrounds, it can make it difficult to segment the region of interest for the identification task. Since the limited sample size for trees in our study was insufficient for statistical analyses, future plot-based studies should further investigate how identification accuracy varies with image covariates or botanical factors, using an appropriate sampling design (e.g. larger plots in forested sites).

Although score fusion may lower the overall confidence of correct identifications after combining perspectives, if a single perspective produces an erroneous identification with high confidence, summing scores from multiple perspectives helps dilute its influence, making incorrect suggestions less dominant. In 2023, plant growth and development in grassland sites were impacted by precipitation levels that were less than half of the 30-year average (Alberta Climate Information System 2024). This limited photography of fruiting and flowering plants, including graminoids. For example, 74% of the graminoid images we collected featured only vegetative structures. In such challenging field conditions, the multiple perspective combination approach plays a crucial role in improving identification accuracy. Rzanny *et al*. (2022) found that, for most species they studied in Poaceae, the use of a single to a few fused perspectives achieved reliable identification accuracy rather than combining all perspectives available, and that the best perspectives were species-specific. In this study, we did not evaluate which perspective combinations achieved high accuracy across different growth forms or species, as we had a varied and imbalanced representation of perspectives per individual as well as species. However, it would be worthwhile to explore the best image combinations for each growth form in the future and see if those combinations can be applied to increase the accuracy of visually similar species groups.

As in this study, mobile apps may not be capable of precise species-level identification in plot-based surveys. Accurate identification can be equally challenging for both artificial intelligence and humans (Munzi *et al*. 2023). For this study, plant taxonomists had to complete their identifications in the laboratory, refining species lists established during fieldwork, suggesting that we should be cautious when evaluating mobile apps. If even human experts find it challenging, it is not fair to penalize AI models for low accuracy. Also, the concept of ‘species’ is inherently complex, as plant classification is a human-defined process based on subtle and sometimes subjective traits (Luckow 1995, Rieseberg *et al*. 2006). Because species boundaries vary across plant groups and can be unclear even to experts, automated applications will continue to face challenges in accurately recognizing some plant species (Scholten 2025). Constructing a visual taxonomy, as in this study for such species complexes, is one way to address the challenges in interpreting species labels from mobile apps.

Genus/family-level identification, or grouping difficult taxa into complexes or aggregates, is a widely accepted pragmatic compromise in ecological monitoring and field surveys when species-level identification is not feasible in the field due to visual similarity or observer limitations (inexperienced field technicians, etc.) (Bevilacqua *et al*. 2012, Rosser 2017, Tälle *et al*. 2023). This approach ensures the reliability of data for biodiversity metrics, particularly when non-experts participate, as it enables reliable data collection at practical resolutions without requiring expert botanical skills. These data can still capture meaningful community patterns and support initial conservation planning, as such grouped data are preferable to having no data at all.

Sometimes, apps like Flora Incognita consider certain visually similar species as aggregates, recognizing that they are typically indistinguishable in field settings. This approach is also applied to taxonomically complex genera like *Rubus*, *Taraxacum*, and *Pilosella* (Rzanny *et al*. 2024). Therefore, it is important to revise the taxonomy before analysing app accuracy. For example, Rzanny *et al*. (2024) reanalysed the test images used by Hart *et al*. (2023) for the Flora Incognita app and found that the reported accuracy improved from 94.4% to 98.8% after correcting for errors in ground truth and taxonomic discrepancies.

Image recognition technology is continuously advancing, leading to improvements in model performance. For example, Jones and Jones (2025) reported a 20% increase in the identification accuracy of mobile apps between 2020 and 2023. Mobile apps are continuously retrained once a significant amount of new and manually reviewed (Flora Incognita) or research-grade observation (iNaturalist) data are available (Mäder *et al*. 2021, López-Guillén *et al*. 2024). In addition to annual training, the CNN model of iNaturalist undergoes monthly model updates with data accuracy assessments, allowing quick learning and iteration on accuracy improvements (https://www.inaturalist.org/pages/computer_vision_demo). Therefore, the accuracy presented in our study represents a snapshot of mobile app performance at the time of testing. Moreover, plant identification apps suffer from spatial biases in their training data, as most observations in training datasets originate from Europe and North America, which limits their generalizability to diverse regions such as tropical rainforests (Goëau *et al*., unpublished data, https://arxiv.org/pdf/2509.18705; Mason *et al*. 2025). Therefore, the results of this study and similar studies from temperate regions may not be reliably transferred to the most diverse ecosystems (e.g. tropical rainforests), where reference data and taxonomic expertise are scarce.

### Recommendations

While high-quality, well-focused images depicting reproductive structures are important for accuracy, we also recommend using multiple images per individual for score fusion. Fusion improves identification accuracy even when reproductive structures or high-quality images are not available. Combining multiple images for identification has already been implemented in Flora Incognita, and this method could also be incorporated into iNaturalist. An additional feature that would benefit both apps is prompting the user (either directly in the field or upon image upload) to define the region of interest, to better focus on small species or features for perspective fusion. To use mobile applications for surveying and monitoring applications, we need to understand the trade-offs between effort and information gain for accurate species identification. For example, if average-quality images perform as well as high-quality images, then investing additional effort in capturing high-quality images may not significantly improve identification accuracy. Moreover, rather than using as many image perspectives as possible for perspective combination, emphasis should be placed on selecting a few informative perspectives that can yield more reliable results. Knowing that kind of information is important in surveying and monitoring efforts that are time and resource-limited (no. of photographers, etc.), in contrast to our study, which is time-unlimited and had multiple observers to collect images.

While most AI research in botany has focused on improving plant identification accuracy by addressing errors and biases, the real promise of photo surveys has just begun to be explored. Botanists are shifting towards training and applying deep learning models beyond species identification. Additional data that can be acquired from photo surveys include hidden phenotypic variation, trait data, and environmental data, thereby contributing directly to species delimitation, biogeography, ecology, and evolutionary inference (Karbstein *et al*. 2024, Mason *et al*. 2025, Lu *et al*. 2026).

## Supplementary Material

plag020_Supplementary_Data

## Data Availability

The image library analysed, datasheets, and R codes are available online at https://doi.org/10.5683/SP3/7YRJ99.
