# Supplementary material for: Evaluating species identification apps as a tool for small plot-based surveys of vascular plants in Alberta, Canada
Source: AoB Plants. 2026 May 8;18(3):plag020. doi: 10.1093/aobpla/plag020 (PMC13394787; doi:10.1093/aobpla/plag020)
Supplement: plag020_Supplementary_Data [file plag020_supplementary_data.docx]

**Supplemental Information**

Table S1. Site locations and dates surveyed.

| Site Type | Site Code | Location (AB, Canada) | Coordinates | Dates Surveyed | |
| --- | --- | --- | --- | --- | --- |
|  |  |  |  | Spring | Summer |
| Grassland Sites | MTEX1 | Mattheis Ranch, Brooks region | 50° 54' 13.87" N, 111° 53' 21.84" W | 9–12 May 2023 | 14–20 July 2023 |
|  | MTCL2 |  | 50° 54' 42.32" N, 111° 53' 33.34" W |  |  |
|  | MTEX3 |  | 50° 53' 5.72" N, 111° 53' 32.66" W |  |  |
| Forest Sites | LFG | Jubilee Forest, Edmonton region | 53° 41' 19.78" N, 113° 19' 16.79" W | 24 May–2 June  2023 | 31 July–10 August 2023 |
|  | WBF | Woodbend Research Forest, Devon region | 53° 23' 29.49" N, 113° 45' 10.28" W |  |  |
|  | EKI | Elk Island National Park | 53° 34' 7.07" N, 112° 50' 36.32" W |  |  |

Table S2. Image perspectives of vascular plants, and criteria for image quality assessment.

| Code for keyword | Category | Description |
| --- | --- | --- |
| EPA | View | Plant full view/entire plant (above) |
| EPS | View | Plant full view/entire plant (side) |
| EPB | View | Entire plant (below)/Canopy |
| LT | Vegetative tissue | Leaf top side |
| LB | Vegetative tissue | Leaf back/lower side |
| NS | Vegetative tissue | Node & stem |
| LIF | Vegetative tissue | Ligule front |
| LIS | Vegetative tissue | Ligule side |
| FF | Reproductive organs | Flower/inflorescence top/frontal |
| FL | Reproductive organs | Flower/inflorescence lateral |
| FTL | Reproductive organs | Spikelet/floret lateral |
| VPR | Reproductive organs | Fruits/pods lateral |
| DM | State | Naturally damaged/diseased |
| PR | State | From previous year |
| SF | Background | Single species focused |
| MF | Background | Multiple species focused |
| V | Background | Vegetation (natural background) |
| NN | Background | Non-natural (palm/white or black sheet/other artificial backgrounds) |
| UO | Background | Images with other objects focused |
| 1 | Image quality | Low quality: Blurry or unclear details/lacking in sharpness, completely out of focus, poor lighting (overexposed/underexposed) |
| 3 | Image quality | Average: Acceptable clarity and sharpness, acceptable lighting and exposure |
| 5 | Image quality | High quality: Excellent clarity and sharpness, well-focused, excellent lighting and exposure |


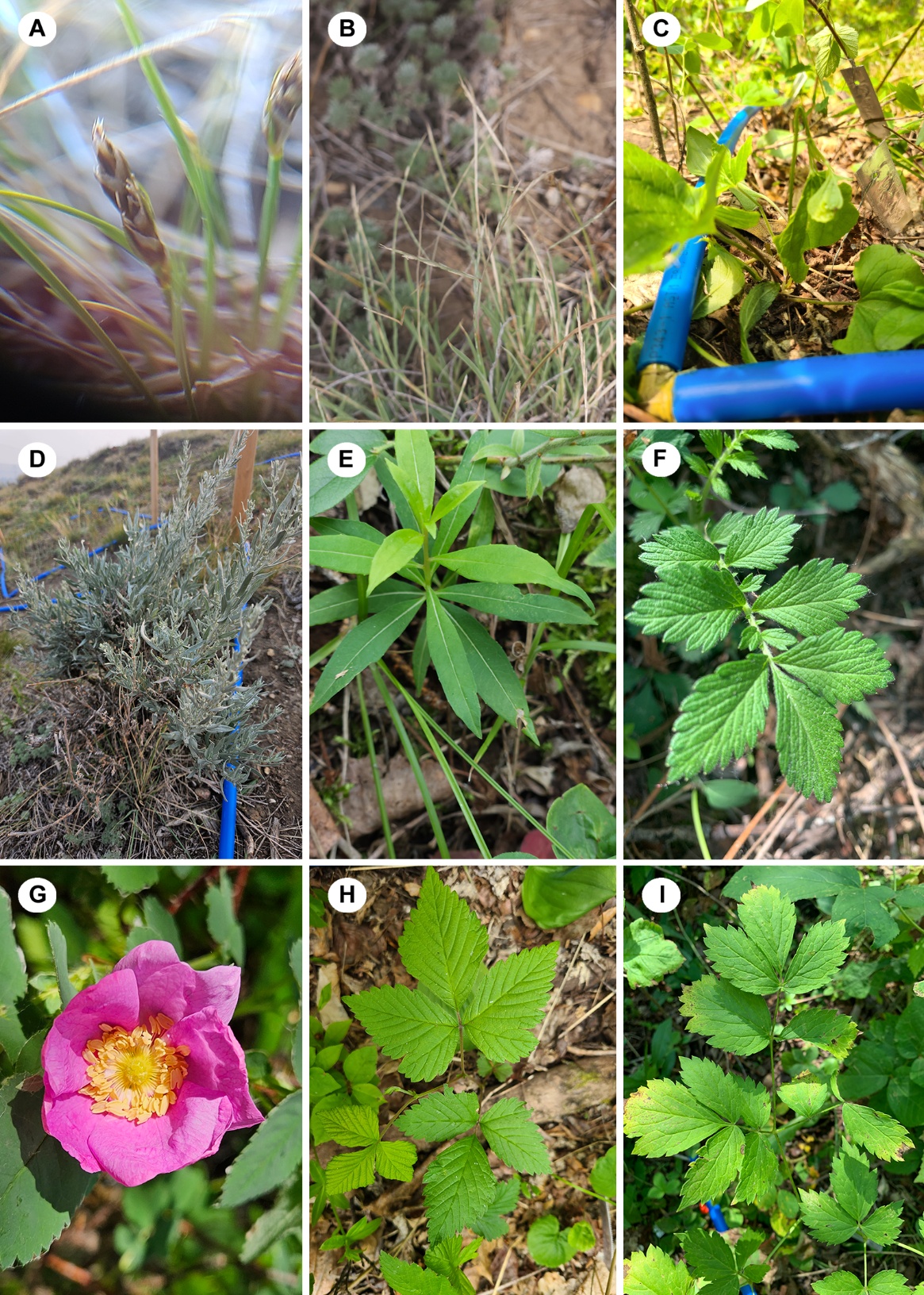


Figure S1. Examples of some low (A-C), average (D-F) and high (G-I) quality images with respective keyword tags (see Table S2): (A) *Carex duriuscula* (SF, V, FL, FTL); (B) *Muhlenbergia paniculata* (FL, V, SF); (C) *Viola canadensis* (DM, UO, V, EPS, MF); (D) *Artemisia cana* (V, SF, EPS, UO); (E) *Chamaenerion angustifolium* (LT, V, SF); (F) *Agrimonia striata* (SF, V, LT); (G) *Rosa acicularis* (V, SF, FF), (H) *Rubus pubescens* (SF, V, EPA); (I) *Actaea rubra* (LT, V, SF).

Table S3. Key additional metadata embedded in existing image metadata.

| IPTC Core | Assigned to |
| --- | --- |
| Headline- | Taxon |
| Creator- | Photographer |
| Description- | Habitat (Grassland/Forest) |
| Sublocation- | Site (MTEX1/MTCL2/MTEX3 /WBF/LFG/EKI) |
| Title | Quadrat |
| Job identifier | Voucher No. (if available) |
| Keywords | Perspectives (refer Table S1 above) |

Table S4. Visual groups defined in our study based on names from experts, Flora Incognita and iNaturalist. All other species were left at species level (see Table S8).

| Species | Visual Group | Family | Description |
| --- | --- | --- | --- |
| *Bromus carinatus* | *Bromus sitchensis* | Poaceae | Complex of species. |
| *Bromus sitchensis* |  |  |  |
| *Calamagrostis inexpansa* | *Calamagrostis stricta* | Poaceae | *Calamagrostis inexpansa is* now considered a subspecies of stricta. |
| *Calamagrostis stricta* |  | Poaceae |  |
| *Campanula alaskana* | *Campanula rotundifolia complex* | Campanulaceae | Essentially visually identical, range is used to distinguish them. Numerous recent taxonomic changes. |
| *Campanula giesekiana* |  |  |  |
| *Campanula intercedens* |  |  |  |
| *Campanula petiolate* |  |  |  |
| *Campanula rotundifolia* |  |  |  |
| *Carex divulsa* | *Carex divulsa/Carex leersii* | Cyperaceae | *C. leersii* is also treated as a subspecies of *C. divulsa*. |
| *Carex leersii* |  |  |  |
| *Carex kelloggii* | *Carex lenticularis/kelloggii* | Cyperaceae | *C. kelloggii* is a species split from C. *lenticularis.* |
| *Carex lenticularis* |  |  |  |
| *Chenopodium album* | *Chenopodium album/Chenopodium berlandieri* | Chenopodiaceae | Need to examine seeds, leaves identical. |
| *Chenopodium berlandieri* |  |  |  |
| *Cornus alba* | *Cornus sericea* | Cornaceae | One subspecies of *C. alba* is syn for sericea, *C*. *alba* appears to be a european species visually similar. |
| *Cornus alba agg.* |  |  |  |
| *Cornus sericea* |  |  |  |
| *Koeleria macrantha* | *Koeleria macrantha/Koeleria pyramidata* | Poaceae | *Koeleria pyramidata* is visually nearly identical and the name has been occasionally misapplied. *K*. *pyramidata* is the European equivalent. |
| *Koeleria pyramidata* |  |  |  |
| *Koeleria pyramidata agg.* |  |  |  |
| *Krascheninnikovia ceratoides* | *Krascheninnikovia ceratoides/Krascheninnikovia lanata* | Chenopodiaceae | North American vs European taxonomy. |
| *Krascheninnikovia ceratoides subsp. Lanata* |  |  |  |
| *Krascheninnikovia lanata* |  |  |  |
| *Pulsatilla nuttalliana* | *Pulsatilla nuttalliana/Pulsatilla patens* | Ranunculaceae | Geographically separated populations. |
| *Pulsatilla patens* |  |  |  |
| *Ribes hirtellum* | *Ribes hirtellum/Ribes inerme* | Grossulariaceae | With or without flowers very similar. The only differences are bark colour and slight differences in leaf shape which are very difficult to interpret. |
| *Ribes inerme* |  |  |  |
| *Solidago altissima* | *Solidago altissima/Solidago canadensis/Solidago lepida* | Asteraceae | *Solidago* subsect. Triplinerviae - photo AI analysis will be biased by inaccurate IDs of the following group of species *Solidago altissima/Solidago canadensis/Solidago lepida* used for training databases. |
| *Solidago canadensis* |  |  |  |
| *Solidago lepida* |  |  |  |
| *Thalictrum occidentale* | *Thalictrum occidentale/Thalictrum sparsiflorum/Thalictrum venulosum* | Ranunculaceae | *Thalictrum venulosum, T. sparsiflorum, and T. occidentale* are all visually identical without flowers/fruits. |
| *Thalictrum sparsiflorum* |  |  |  |
| *Thalictrum venulosum* |  |  |  |
| *Thinopyrum elongatum* | *Thinopyrum elongatum/Thinopyrum obtusiflorum* | Poaceae | Synonyms. |
| *Thinopyrum obtusiflorum* |  |  |  |

Table S5. Summary of studies evaluating mobile apps for vascular plant identification (Here we considered identification accuracy at the species level when it is given as a top-1 correct suggestion).

| Study | App | Species Level Accuracy % |
| --- | --- | --- |
| Popp et al (2025) | Flora Incognita | 93 |
|  | FloraID | 90 |
|  | iNaturalist | 67 |
|  | PlantNet | 75 |
| Partel et al (2021) | Flora Incognita | 85.3 |
| Mader et al (2021) | Flora Incognita | 93 |
| Schmidt & Steinecke (2019) | Flora Incognita | 80 |
|  | iNaturalist | 70 |
|  | PlantNet | 76.7 |
| Lüdemann (2020) | Flora Incognita | 87.7 |
|  | PlantNet | 78 |
| Bilyk et al (2020) | PlantNet | 55 |
|  | Flora Incognita | 71 |
| Scholten (2025) | Picture This | 69.4 |
|  | PlantNet | 65.1 |
|  | iNaturalist | 64.5 |
|  | Google Lens | 51.1 |
| Xing et al (2021) | 5 apps (Best performer) | 74.6 |
| Otter et al (2021) | Picture This | 91.1 |
|  | PlantSnap | 62.3 |
|  | PlantNet | 83.1 |
| Rzanny et al (2024) | Flora Incognita | 98.8 |
| Jones & Jones (2025) | Plant id | 71 |
|  | Flora Incognita | 97 |
|  | PlantNet | 87 |
|  | iNaturalist | 61 |
|  | Seek | 69 |
|  | Google Lens | 50 |
| Anyomi (2023) | PlantNet | 79 |
|  | iNaturalist | 44 |
| Hart et al (2023) | PlantNet | 86.6 |
|  | PlantSnap | 46.4 |
|  | LeafSnap | 86.9 |
|  | iNaturalist | 65.6 |
|  | Google Lens | 57 |
| Schmidt et al (2022) | Summary across six apps across spp (leaf)- (Picture This, iNaturalist, Plant Identification, PlantNet, LeafSnap, PlantSnap) | 65.3 |

Table S6. Top-1 ID accuracy of Flora Incognita and iNaturalist at different expert analytical levels (n = number of images tested).

| App | Analytical Level | n | Top 1 Accuracy % | Top 1 Accuracy (Genus) % |
| --- | --- | --- | --- | --- |
| Flora Incognita | Family | 3 | 100.0 | NA |
|  | Order | 1 | 0.0 | NA |
|  | Subgenus | 5 | 0.0 | NA |
|  | Subsection | 17 | 41.2 | NA |
|  | Genus | 183 | 86.3 | NA |
|  | Species | 4488 | 79.2 | 88.4 |
| iNaturalist | Family | 5 | 60.0 | NA |
|  | Order | 4 | 0.0 | NA |
|  | Subgenus | 6 | 33.3 | NA |
|  | Subsection | 20 | 35.0 | NA |
|  | Genus | 200 | 58.5 | NA |
|  | Species | 5040 | 66.5 | 73.7 |

Table S7. Top-1 ID genus-level accuracy of the species not included in Flora Incognita (n = number of images tested).

| Species | n | Genus ID Accuracy (%) |
| --- | --- | --- |
| *Elymus trachycaulus* | 3 | 0.0 |
| *Heracleum maximum* | 22 | 100.0 |
| *Leymus innovatus* | 37 | 0.0 |
| *Muhlenbergia cuspidata* | 81 | 4.9 |
| *Physaria arenosa* | 38 | 76.3 |
| *Piptatheropsis pungens* | 4 | 0.0 |
| *Rubus pubescens* | 158 | 98.7 |

Table S8. Species- and genus-level ID accuracy of vascular plants in Flora Incognita and iNaturalist (n = no. of images tested, NA = Species that are not included in the Flora Incognita app).

| Species | Flora Incognita | | | iNaturalist | | |
| --- | --- | --- | --- | --- | --- | --- |
|  | n | Species | Genus | n | Species | Genus |
| *Acer negundo* | 32 | 100.0 | 100.0 | 32 | 84.4 | 84.4 |
| *Achillea borealis* | 11 | 100.0 | 100.0 | 11 | 81.8 | 100.0 |
| *Actaea rubra* | 61 | 100.0 | 100.0 | 61 | 95.1 | 95.1 |
| *Agrimonia striata* | 13 | 0.0 | 100.0 | 13 | 69.2 | 69.2 |
| *Amelanchier alnifolia* | 50 | 84.0 | 100.0 | 50 | 94.0 | 94.0 |
| *Anemonastrum canadense* | 15 | 93.3 | 93.3 | 15 | 86.7 | 86.7 |
| *Anemone virginiana* | 7 | 100.0 | 100.0 | 7 | 100.0 | 100.0 |
| *Apocynum androsaemifolium* | 13 | 84.6 | 84.6 | 13 | 53.9 | 53.9 |
| *Aralia nudicaulis* | 118 | 98.3 | 98.3 | 118 | 88.1 | 88.1 |
| *Artemisia cana* | 24 | 50.0 | 83.3 | 24 | 62.5 | 70.8 |
| *Artemisia frigida* | 190 | 98.4 | 100.0 | 190 | 92.1 | 93.7 |
| *Artemisia ludoviciana* | 118 | 89.8 | 94.9 | 125 | 44.8 | 56.8 |
| *Astragalus agrestis* | 24 | 54.2 | 100.0 | 24 | 41.7 | 66.7 |
| *Astragalus missouriensis* | 27 | 85.2 | 100.0 | 27 | 70.4 | 92.6 |
| *Boechera retrofracta* | 22 | 40.9 | 40.9 | 22 | 45.5 | 45.5 |
| *Bouteloua gracilis* | 102 | 73.5 | 74.5 | 107 | 14.0 | 14.0 |
| *Bromus ciliatus* | 15 | 33.3 | 40.0 | 17 | 29.4 | 35.3 |
| *Calamagrostis canadensis* | 22 | 0.0 | 0.0 | 27 | 0.0 | 0.0 |
| *Calamagrostis stricta* | 10 | 0.0 | 0.0 | 12 | 0.0 | 0.0 |
| *Campanula rotundifolia* | 7 | 85.7 | 85.7 | 7 | 0.0 | 0.0 |
| *Carex deweyana* | 8 | 50.0 | 100.0 | 9 | 33.3 | 100.0 |
| *Carex duriuscula* | 73 | 27.4 | 74.0 | 90 | 42.2 | 85.6 |
| *Carex filifolia* | 85 | 84.7 | 89.4 | 87 | 75.9 | 85.1 |
| *Carex inops* | 18 | 11.1 | 77.8 | 20 | 5.0 | 75.0 |
| *Carex peckii* | 21 | 66.7 | 90.5 | 25 | 40.0 | 92.0 |
| *Carex siccata* | 7 | 71.4 | 85.7 | 7 | 57.1 | 85.7 |
| *Cerastium arvense* | 19 | 100.0 | 100.0 | 19 | 79.0 | 79.0 |
| *Chamaenerion angustifolium* | 15 | 80.0 | 80.0 | 19 | 89.5 | 89.5 |
| *Comandra umbellata* | 45 | 95.6 | 95.6 | 46 | 69.6 | 69.6 |
| *Cornus canadensis* | 88 | 90.9 | 100.0 | 88 | 93.2 | 93.2 |
| *Cornus sericea* | 16 | 93.8 | 100.0 | 16 | 62.5 | 75.0 |
| *Corylus cornuta* | 50 | 100.0 | 100.0 | 50 | 88.0 | 90.0 |
| *Dalea purpurea* | 18 | 100.0 | 100.0 | 18 | 77.8 | 88.9 |
| *Elymus trachycaulus* | NA | NA | NA | 5 | 0.0 | 0.0 |
| *Equisetum hyemale* | 3 | 100.0 | 100.0 | 3 | 100.0 | 100.0 |
| *Equisetum laevigatum* | 45 | 71.1 | 97.8 | 45 | 77.8 | 80.0 |
| *Equisetum pratense* | 5 | 100.0 | 100.0 | 5 | 60.0 | 100.0 |
| *Erigeron caespitosus* | 28 | 10.7 | 32.1 | 40 | 35.0 | 40.0 |
| *Erigeron philadelphicus* | 5 | 0.0 | 100.0 | 5 | 40.0 | 80.0 |
| *Eriogonum flavum* | 24 | 100.0 | 100.0 | 24 | 95.8 | 95.8 |
| *Erysimum inconspicuum* | 8 | 0.0 | 75.0 | 8 | 0.0 | 12.5 |
| *Escobaria vivipara* | 41 | 95.1 | 100.0 | 41 | 95.1 | 100.0 |
| *Fragaria vesca* | 26 | 92.3 | 92.3 | 26 | 80.8 | 88.5 |
| *Fragaria virginiana* | 61 | 96.7 | 98.4 | 61 | 98.4 | 98.4 |
| *Fraxinus pennsylvanica* | 27 | 77.8 | 92.6 | 27 | 74.1 | 81.5 |
| *Galium boreale* | 55 | 98.2 | 98.2 | 55 | 78.2 | 81.8 |
| *Galium triflorum* | 59 | 98.3 | 98.3 | 59 | 98.3 | 98.3 |
| *Gutierrezia sarothrae* | 68 | 100.0 | 100.0 | 69 | 82.6 | 82.6 |
| *Halenia deflexa* | 9 | 100.0 | 100.0 | 9 | 100.0 | 100.0 |
| *Hedysarum boreale* | 16 | 68.8 | 75.0 | 16 | 18.8 | 31.3 |
| *Heracleum maximum* | NA | NA | NA | 22 | 72.7 | 72.7 |
| *Hesperostipa comata* | 199 | 50.3 | 51.3 | 236 | 14.8 | 17.4 |
| *Heterotheca villosa* | 23 | 91.3 | 95.7 | 24 | 70.8 | 70.8 |
| *Hymenoxys richardsonii* | 29 | 100.0 | 100.0 | 29 | 100.0 | 100.0 |
| *Juncus balticus* | 17 | 29.4 | 52.9 | 21 | 33.3 | 38.1 |
| *Juniperus horizontalis* | 41 | 97.6 | 100.0 | 41 | 100.0 | 100.0 |
| *Koeleria macrantha/Koeleria pyramidata* | 102 | 67.7 | 68.6 | 115 | 20.0 | 25.2 |
| *Lathyrus ochroleucus* | 67 | 88.1 | 98.5 | 67 | 91.0 | 95.5 |
| *Lathyrus venosus* | 18 | 77.8 | 100.0 | 18 | 77.8 | 88.9 |
| *Leymus innovatus* | NA | NA | NA | 49 | 30.6 | 30.6 |
| *Liatris punctata* | 12 | 75.0 | 75.0 | 12 | 25.0 | 25.0 |
| *Linnaea borealis* | 39 | 100.0 | 100.0 | 39 | 94.9 | 94.9 |
| *Linum lewisii* | 24 | 75.0 | 83.3 | 27 | 51.9 | 63.0 |
| *Lonicera dioica* | 51 | 64.7 | 96.1 | 51 | 66.7 | 86.3 |
| *Lonicera involucrata* | 21 | 100.0 | 100.0 | 21 | 71.4 | 95.2 |
| *Lygodesmia juncea* | 37 | 75.7 | 75.7 | 47 | 48.9 | 48.9 |
| *Maianthemum canadense* | 106 | 86.8 | 100.0 | 106 | 82.1 | 88.7 |
| *Maianthemum stellatum* | 44 | 97.7 | 97.7 | 44 | 88.6 | 88.6 |
| *Mertensia paniculata* | 27 | 100.0 | 100.0 | 27 | 96.3 | 96.3 |
| *Mitella nuda* | 48 | 97.9 | 97.9 | 48 | 95.8 | 95.8 |
| *Moehringia lateriflora* | 11 | 100.0 | 100.0 | 11 | 90.9 | 90.9 |
| *Muhlenbergia cuspidata* | NA | NA | NA | 98 | 4.1 | 17.4 |
| *Nassella viridula* | 31 | 61.3 | 61.3 | 40 | 50.0 | 50.0 |
| *Opuntia polyacantha* | 40 | 70.0 | 100.0 | 40 | 87.5 | 97.5 |
| *Oryzopsis asperifolia* | 31 | 77.4 | 77.4 | 37 | 64.9 | 64.9 |
| *Packera cana* | 17 | 88.2 | 88.2 | 17 | 76.5 | 76.5 |
| *Paronychia sessiliflora* | 19 | 100.0 | 100.0 | 19 | 100.0 | 100.0 |
| *Pascopyrum smithii* | 57 | 15.8 | 15.8 | 69 | 20.3 | 20.3 |
| *Petasites frigidus* | 22 | 95.5 | 95.5 | 23 | 87.0 | 87.0 |
| *Phlox hoodii* | 85 | 96.5 | 96.5 | 85 | 98.8 | 98.8 |
| *Physaria arenosa* | NA | NA | NA | 36 | 83.3 | 86.1 |
| *Picea glauca* | 25 | 44.0 | 92.0 | 26 | 53.9 | 61.5 |
| *Piptatheropsis pungens* | NA | NA | NA | 6 | 66.7 | 66.7 |
| *Plantago major* | 14 | 100.0 | 100.0 | 14 | 92.9 | 92.9 |
| *Poa pratensis* | 2 | 50.0 | 50.0 | 6 | 0.0 | 0.0 |
| *Populus balsamifera* | 4 | 100.0 | 100.0 | 4 | 75.0 | 75.0 |
| *Populus tremuloides* | 56 | 96.4 | 98.2 | 56 | 89.3 | 89.3 |
| *Prosartes trachycarpa* | 27 | 96.3 | 96.3 | 27 | 100.0 | 100.0 |
| *Prunus pensylvanica* | 22 | 72.7 | 81.8 | 23 | 52.2 | 56.5 |
| *Prunus virginiana* | 50 | 86.0 | 98.0 | 51 | 52.9 | 56.9 |
| *Pulsatilla nuttalliana/Pulsatilla patens* | 43 | 86.1 | 90.7 | 43 | 74.4 | 79.1 |
| *Pyrola asarifolia* | 60 | 90.0 | 98.3 | 60 | 83.3 | 86.7 |
| *Ranunculus abortivus* | 11 | 100.0 | 100.0 | 11 | 45.5 | 72.7 |
| *Ribes hirtellum/Ribes inerme* | 16 | 6.3 | 100.0 | 16 | 0.0 | 100.0 |
| *Ribes triste* | 10 | 100.0 | 100.0 | 10 | 100.0 | 100.0 |
| *Rosa acicularis* | 105 | 82.9 | 100.0 | 105 | 70.5 | 85.7 |
| *Rosa arkansana* | 104 | 71.2 | 98.1 | 105 | 67.6 | 86.7 |
| *Rosa woodsii* | 27 | 14.8 | 100.0 | 27 | 33.3 | 88.9 |
| *Rubus idaeus* | 87 | 86.2 | 95.4 | 87 | 64.4 | 82.8 |
| *Rubus pubescens* | NA | NA | NA | 158 | 89.2 | 95.6 |
| *Sanicula marilandica* | 23 | 100.0 | 100.0 | 23 | 100.0 | 100.0 |
| *Selaginella densa* | 43 | 100.0 | 100.0 | 43 | 100.0 | 100.0 |
| *Solidago decemflora* | 4 | 0.0 | 75.0 | 4 | 0.0 | 0.0 |
| *Solidago gigantea* | 60 | 58.3 | 76.7 | 62 | 46.8 | 67.7 |
| *Sphaeralcea coccinea* | 69 | 97.1 | 97.1 | 70 | 81.4 | 81.4 |
| *Sporobolus rigidus* | 118 | 55.9 | 66.1 | 123 | 80.5 | 80.5 |
| *Stachys pilosa* | 32 | 0.0 | 56.3 | 32 | 65.6 | 68.8 |
| *Stellaria longifolia* | 6 | 66.7 | 83.3 | 6 | 83.3 | 83.3 |
| *Symphoricarpos albus* | 70 | 98.6 | 100.0 | 71 | 46.5 | 62.0 |
| *Symphyotrichum ciliolatum* | 58 | 70.7 | 77.6 | 59 | 67.8 | 78.0 |
| *Trifolium pratense* | 1 | 0.0 | 0.0 | 1 | 0.0 | 0.0 |
| *Vaccinium myrtilloides* | 63 | 98.4 | 98.4 | 63 | 92.1 | 93.7 |
| *Viburnum edule* | 56 | 100.0 | 100.0 | 56 | 94.6 | 96.4 |
| *Viburnum opulus* | 36 | 94.4 | 97.2 | 36 | 80.6 | 80.6 |
| *Vicia americana* | 39 | 76.9 | 97.4 | 39 | 64.1 | 74.4 |
| *Viola adunca* | 11 | 63.6 | 100.0 | 11 | 18.2 | 90.9 |
| *Viola canadensis* | 23 | 95.7 | 100.0 | 23 | 82.6 | 91.3 |
| *Viola renifolia* | 31 | 77.4 | 96.8 | 31 | 83.9 | 96.8 |
| *Xanthisma spinulosum* | 38 | 86.8 | 86.8 | 39 | 69.2 | 71.8 |

Table S9. Growth form descriptions.

| Growth Form | Definition |
| --- | --- |
| Tree | A perennial woody plant with a single main stem generally branching at some height above the ground to form a distinct crown. Typically, have a minimum height of 3-6 m at maturity, though this can vary depending on the specific classification system used. |
| Shrub | A multi-stemmed woody perennial plant, both evergreen and deciduous. Shrubs typically have several stems arising at or near the ground, branches may be erect and spreading or prostrate (e.g. *Juniperus horizontalis*). A reporting break is often made between tall shrubs (≥ 2 m in height) and low shrubs (< 2 m in height). |
| Herb | A vascular plant that does not develop persistent woody tissue above ground. Herbs have soft, non-woody stems that generally die back to the ground at the end of each growing season. |
| Graminoid | A herbaceous plant with a grass-like morphology, characterized by elongated culms with long, blade-like leaves. This group primarily includes members of the families Poaceae (grasses), Cyperaceae (sedges), and Juncaceae (rushes). |

Table S10. Summary table of application-reported confidence in the first suggestion according to growth forms (Flora Incognita on a 0–1 scale and iNaturalist on a 0–100 scale).

| App | Growth Form | Confidence in the first suggestion, where that suggestion is correct | | | | | Confidence in the first suggestion, where that suggestion is incorrect | | | | |
| --- | --- | --- | --- | --- | --- | --- | --- | --- | --- | --- | --- |
|  |  | Mean | Median | Min | Max | SD | Mean | Median | Min | Max | SD |
| Flora Incognita (Before Score Fusion) | Graminoid | 0.737 | 0.806 | 0.206 | 1.000 | 0.236 | 0.481 | 0.435 | 0.201 | 0.995 | 0.206 |
|  | Herb | 0.928 | 0.989 | 0.209 | 1.000 | 0.137 | 0.576 | 0.552 | 0.202 | 0.998 | 0.228 |
|  | Shrub | 0.909 | 0.970 | 0.277 | 0.998 | 0.137 | 0.668 | 0.651 | 0.202 | 0.997 | 0.223 |
|  | Tree | 0.922 | 0.984 | 0.362 | 0.999 | 0.134 | 0.624 | 0.649 | 0.214 | 0.912 | 0.208 |
| Flora Incognita (After Score Fusion) | Graminoid | 0.658 | 0.636 | 0.230 | 1.021 | 0.212 | 0.488 | 0.430 | 0.204 | 0.995 | 0.215 |
|  | Herb | 0.899 | 0.978 | 0.217 | 1.334 | 0.152 | 0.551 | 0.523 | 0.212 | 0.997 | 0.203 |
|  | Shrub | 0.875 | 0.937 | 0.304 | 1.423 | 0.155 | 0.629 | 0.567 | 0.250 | 0.995 | 0.219 |
|  | Tree | 0.910 | 0.958 | 0.566 | 0.997 | 0.113 | 0.554 | 0.491 | 0.256 | 0.852 | 0.237 |
| iNaturalist (Before Score Fusion) | Graminoid | 64.75 | 73.01 | 2.90 | 99.96 | 30.38 | 24.39 | 17.54 | 1.16 | 98.74 | 19.85 |
|  | Herb | 77.58 | 90.23 | 1.68 | 100.00 | 26.17 | 27.42 | 20.43 | 1.39 | 99.60 | 21.17 |
|  | Shrub | 72.32 | 79.68 | 3.63 | 99.89 | 25.24 | 39.57 | 33.98 | 2.28 | 98.45 | 24.66 |
|  | Tree | 67.37 | 74.77 | 5.10 | 99.61 | 28.89 | 20.79 | 18.96 | 3.35 | 82.22 | 17.16 |
| iNaturalist (After Score Fusion) | Graminoid | 60.87 | 57.11 | 12.55 | 98.81 | 24.37 | 31.96 | 25.93 | 3.23 | 92.51 | 18.71 |
|  | Herb | 73.24 | 77.32 | 13.05 | 110.55 | 22.65 | 46.89 | 34.71 | 6.58 | 99.89 | 31.07 |
|  | Shrub | 64.95 | 66.23 | 12.85 | 99.65 | 22.11 | 45.93 | 41.01 | 18.28 | 93.75 | 20.18 |
|  | Tree | 65.24 | 68.37 | 24.95 | 90.75 | 18.00 | 25.82 | 23.27 | 15.89 | 44.15 | 10.07 |

Table S11. ID accuracy by image covariates for different growth forms in Flora Incognita and iNaturalist (n = total number of images).

| Growth Form | Image covariate | Flora Incognita | | | iNaturalist | | |
| --- | --- | --- | --- | --- | --- | --- | --- |
|  |  | n | Correct | Accuracy (%) | n | Correct | Accuracy (%) |
| Graminoid | Not Reproductive | 636 | 275 | 43.2 | 906 | 178 | 19.6 |
|  | Reproductive | 284 | 215 | 75.7 | 294 | 205 | 69.7 |
|  | Low quality | 189 | 86 | 45.5 | 269 | 62 | 23.0 |
|  | Average quality | 549 | 302 | 55.0 | 696 | 213 | 30.6 |
|  | High quality | 182 | 102 | 56.0 | 235 | 108 | 46.0 |
|  | Single species focused | 830 | 458 | 55.2 | 1089 | 357 | 32.8 |
|  | Multiple species focused | 90 | 32 | 35.6 | 111 | 26 | 23.4 |
| Herb | Not Reproductive | 2058 | 1750 | 85.0 | 2226 | 1719 | 77.2 |
|  | Reproductive | 491 | 460 | 93.7 | 590 | 515 | 87.3 |
|  | Low quality | 255 | 200 | 78.4 | 283 | 179 | 63.3 |
|  | Average quality | 1250 | 1092 | 87.4 | 1369 | 1061 | 77.5 |
|  | High quality | 1044 | 918 | 87.9 | 1164 | 994 | 85.4 |
|  | Single species focused | 2196 | 1899 | 86.5 | 2427 | 1917 | 79.0 |
|  | Multiple species focused | 353 | 311 | 88.1 | 389 | 317 | 81.5 |
| Shrub | Not Reproductive | 813 | 684 | 84.1 | 817 | 582 | 71.2 |
|  | Reproductive | 62 | 48 | 77.4 | 62 | 41 | 66.1 |
|  | Low quality | 55 | 46 | 83.6 | 58 | 34 | 58.6 |
|  | Average quality | 346 | 288 | 83.2 | 346 | 240 | 69.4 |
|  | High quality | 474 | 398 | 84.0 | 475 | 349 | 73.5 |
|  | Single species focused | 779 | 652 | 83.7 | 782 | 558 | 71.4 |
|  | Multiple species focused | 96 | 80 | 83.3 | 97 | 65 | 67.0 |
| Tree | Not Reproductive | 143 | 121 | 84.6 | 144 | 113 | 78.5 |
|  | Reproductive | 1 | 1 | 100.0 | 1 | 1 | 100.0 |
|  | Low quality | 1 | 1 | 100.0 | 1 | 1 | 100.0 |
|  | Average quality | 39 | 33 | 84.6 | 40 | 31 | 77.5 |
|  | High quality | 104 | 88 | 84.6 | 104 | 82 | 78.8 |
|  | Single species focused | 129 | 113 | 87.6 | 130 | 104 | 80.0 |
|  | Multiple species focused | 15 | 9 | 60.0 | 15 | 10 | 66.7 |

Table S12. ID accuracy summary by species before and after applying perspective combination in both apps. In the columns “Overall accuracy change (%)”, accuracy change values are color coded as blue-accuracy increased; red-accuracy decreased; black-no accuracy change, NA-Species not trained in Flora Incognita. Accuracy before fusion is the average across all images of each species.

| Species | Total no. of individuals | Flora Incognita | | | iNaturalist | | |
| --- | --- | --- | --- | --- | --- | --- | --- |
|  |  | Accuracy (%) | Accuracy after score fusion (%) | Overall accuracy change (%) | Accuracy (%) | Accuracy after score fusion | Overall accuracy change (%) |
| *Acer negundo* | 8 | 100.0 | 100.0 | 0.0 | 84.4 | 87.5 | **3.1** |
| *Achillea borealis* | 3 | 100.0 | 100.0 | 0.0 | 81.8 | 100.0 | **18.2** |
| *Actaea rubra* | 11 | 100.0 | 100.0 | 0.0 | 95.1 | 100.0 | **4.9** |
| *Agrimonia striata* | 4 | 0.0 | 0.0 | 0.0 | 69.2 | 75.0 | **5.8** |
| *Amelanchier alnifolia* | 13 | 84.0 | 92.3 | **8.3** | 94.0 | 100.0 | **6.0** |
| *Anemonastrum canadense* | 4 | 93.3 | 100.0 | **6.7** | 86.7 | 100.0 | **13.3** |
| *Anemone virginiana* | 1 | 100.0 | 100.0 | 0.0 | 100.0 | 100.0 | 0.0 |
| *Apocynum androsaemifolium* | 3 | 84.6 | 100.0 | **15.4** | 53.9 | 66.7 | **12.8** |
| *Aralia nudicaulis* | 27 | 98.3 | 100.0 | **1.7** | 88.1 | 96.3 | **8.2** |
| *Artemisia cana* | 6 | 50.0 | 50.0 | 0.0 | 62.5 | 83.3 | **20.8** |
| *Artemisia frigida* | 47 | 98.4 | 100.0 | **1.6** | 92.1 | 97.9 | **5.8** |
| *Artemisia ludoviciana* | 21 | 89.8 | 85.7 | **-4.1** | 44.8 | 57.1 | **12.3** |
| *Astragalus agrestis* | 7 | 54.2 | 57.1 | **3.0** | 41.7 | 28.6 | **-13.1** |
| *Astragalus missouriensis* | 9 | 85.2 | 88.9 | **3.7** | 70.4 | 77.8 | **7.4** |
| *Boechera retrofracta* | 6 | 40.9 | 50.0 | **9.1** | 45.5 | 50.0 | **4.6** |
| *Bouteloua gracilis* | 31 | 73.5 | 83.9 | **10.3** | 14.0 | 29.0 | **15.0** |
| *Bromus ciliatus* | 4 | 33.3 | 25.0 | **-8.3** | 29.4 | 25.0 | **-4.4** |
| *Calamagrostis canadensis* | 7 | 0.0 | 0.0 | 0.0 | 0.0 | 0.0 | 0.0 |
| *Calamagrostis stricta* | 2 | 0.0 | 0.0 | 0.0 | 0.0 | 0.0 | 0.0 |
| *Campanula rotundifolia* | 2 | 85.7 | 100.0 | **14.3** | 0.0 | 0.0 | 0.0 |
| *Carex deweyana* | 1 | 50.0 | 0.0 | **-50.0** | 33.3 | 100.0 | **66.7** |
| *Carex duriuscula* | 29 | 27.4 | 27.6 | **0.2** | 42.2 | 48.3 | **6.1** |
| *Carex filifolia* | 24 | 84.7 | 87.5 | **2.8** | 75.9 | 70.8 | **-5.0** |
| *Carex inops* | 6 | 11.1 | 0.0 | **-11.1** | 5.0 | 0.0 | **-5.0** |
| *Carex peckii* | 7 | 66.7 | 85.7 | **19.0** | 40.0 | 42.9 | **2.9** |
| *Carex siccata* | 2 | 71.4 | 100.0 | **28.6** | 57.1 | 100.0 | **42.9** |
| *Cerastium arvense* | 3 | 100.0 | 100.0 | 0.0 | 79.0 | 100.0 | **21.1** |
| *Chamaenerion angustifolium* | 5 | 80.0 | 60.0 | **-20.0** | 89.5 | 100.0 | **10.5** |
| *Comandra umbellata* | 9 | 95.6 | 100.0 | **4.4** | 69.6 | 77.8 | **8.2** |
| *Cornus canadensis* | 23 | 90.9 | 100.0 | **9.1** | 93.2 | 95.7 | **2.5** |
| *Cornus sericea* | 4 | 93.8 | 100.0 | **6.3** | 62.5 | 75.0 | **12.5** |
| *Corylus cornuta* | 13 | 100.0 | 100.0 | 0.0 | 88.0 | 92.3 | **4.3** |
| *Dalea purpurea* | 4 | 100.0 | 100.0 | 0.0 | 77.8 | 75.0 | **-2.8** |
| *Elymus trachycaulus* | 1 | NA | NA | NA | 0.0 | 0.0 | 0.0 |
| *Equisetum hyemale* | 1 | 100.0 | 100.0 | 0.0 | 100.0 | 100.0 | 0.0 |
| *Equisetum laevigatum* | 6 | 71.1 | 83.3 | **12.2** | 77.8 | 83.3 | **5.6** |
| *Equisetum pratense* | 2 | 100.0 | 100.0 | 0.0 | 60.0 | 100.0 | **40.0** |
| *Erigeron caespitosus* | 12 | 10.7 | 25.0 | **14.3** | 35.0 | 41.7 | **6.7** |
| *Erigeron philadelphicus* | 1 | 0.0 | 0.0 | 0.0 | 40.0 | 100.0 | **60.0** |
| *Eriogonum flavum* | 5 | 100.0 | 100.0 | 0.0 | 95.8 | 100.0 | **4.2** |
| *Erysimum inconspicuum* | 2 | 0.0 | 0.0 | 0.0 | 0.0 | 0.0 | 0.0 |
| *Escobaria vivipara* | 14 | 95.1 | 92.9 | **-2.3** | 95.1 | 92.9 | **-2.3** |
| *Fragaria vesca* | 7 | 92.3 | 85.7 | **-6.6** | 80.8 | 85.7 | **4.9** |
| *Fragaria virginiana* | 19 | 96.7 | 100.0 | **3.3** | 98.4 | 94.7 | **-3.6** |
| *Fraxinus pennsylvanica* | 7 | 77.8 | 85.7 | **7.9** | 74.1 | 85.7 | **11.6** |
| *Galium boreale* | 17 | 98.2 | 100.0 | **1.8** | 78.2 | 82.4 | **4.2** |
| *Galium triflorum* | 13 | 98.3 | 100.0 | **1.7** | 98.3 | 100.0 | **1.7** |
| *Gutierrezia sarothrae* | 15 | 100.0 | 100.0 | 0.0 | 82.6 | 80.0 | **-2.6** |
| *Halenia deflexa* | 2 | 100.0 | 100.0 | 0.0 | 100.0 | 100.0 | 0.0 |
| *Hedysarum boreale* | 3 | 68.8 | 100.0 | **31.3** | 18.8 | 0.0 | **-18.8** |
| *Heracleum maximum* | 6 | NA | NA | NA | 72.7 | 83.3 | **10.6** |
| *Hesperostipa comata* | 48 | 50.3 | 56.3 | **6.0** | 14.8 | 27.1 | **12.3** |
| *Heterotheca villosa* | 3 | 91.3 | 100.0 | **8.7** | 70.8 | 66.7 | **-4.2** |
| *Hymenoxys richardsonii* | 6 | 100.0 | 100.0 | 0.0 | 100.0 | 100.0 | 0.0 |
| *Juncus balticus* | 4 | 29.4 | 25.0 | **-4.4** | 33.3 | 25.0 | **-8.3** |
| *Juniperus horizontalis* | 17 | 97.6 | 94.1 | **-3.4** | 100.0 | 100.0 | 0.0 |
| *Koeleria macrantha/Koeleria pyramidata* | 28 | 67.7 | 75.0 | **7.3** | 20.0 | 32.1 | **12.1** |
| *Lathyrus ochroleucus* | 15 | 88.1 | 86.7 | **-1.4** | 91.0 | 93.3 | **2.3** |
| *Lathyrus venosus* | 5 | 77.8 | 80.0 | **2.2** | 77.8 | 100.0 | **22.2** |
| *Leymus innovatus* | 11 | NA | NA | NA | 30.6 | 27.3 | **-3.3** |
| *Liatris punctata* | 2 | 75.0 | 100.0 | **25.0** | 25.0 | 50.0 | **25.0** |
| *Linnaea borealis* | 12 | 100.0 | 100.0 | 0.0 | 94.9 | 100.0 | **5.1** |
| *Linum lewisii* | 7 | 75.0 | 71.4 | **-3.6** | 51.9 | 57.1 | **5.3** |
| *Lonicera dioica* | 11 | 64.7 | 72.7 | **8.0** | 66.7 | 72.7 | **6.1** |
| *Lonicera involucrata* | 5 | 100.0 | 100.0 | 0.0 | 71.4 | 80.0 | **8.6** |
| *Lygodesmia juncea* | 13 | 75.7 | 76.9 | **1.2** | 48.9 | 61.5 | **12.6** |
| *Maianthemum canadense* | 24 | 86.8 | 87.5 | **0.7** | 82.1 | 95.8 | **13.8** |
| *Maianthemum stellatum* | 6 | 97.7 | 100.0 | **2.3** | 88.6 | 100.0 | **11.4** |
| *Mertensia paniculata* | 4 | 100.0 | 100.0 | 0.0 | 96.3 | 100.0 | **3.7** |
| *Mitella nuda* | 9 | 97.9 | 100.0 | **2.1** | 95.8 | 100.0 | **4.2** |
| *Moehringia lateriflora* | 1 | 100.0 | 100.0 | 0.0 | 90.9 | 100.0 | **9.1** |
| *Muhlenbergia cuspidata* | 18 | NA | NA | NA | 4.1 | 5.6 | **1.5** |
| *Nassella viridula* | 7 | 61.3 | 71.4 | **10.1** | 50.0 | 42.9 | **-7.1** |
| *Opuntia polyacantha* | 13 | 70.0 | 76.9 | **6.9** | 87.5 | 100.0 | **12.5** |
| *Oryzopsis asperifolia* | 10 | 77.4 | 90.0 | **12.6** | 64.9 | 70.0 | **5.1** |
| *Packera cana* | 3 | 88.2 | 100.0 | **11.8** | 76.5 | 100.0 | **23.5** |
| *Paronychia sessiliflora* | 4 | 100.0 | 100.0 | 0.0 | 100.0 | 100.0 | 0.0 |
| *Pascopyrum smithii* | 18 | 15.8 | 12.5 | **-3.3** | 20.3 | 5.6 | **-14.7** |
| *Petasites frigidus* | 10 | 95.5 | 100.0 | **4.6** | 87.0 | 90.0 | **3.0** |
| *Phlox hoodii* | 21 | 96.5 | 95.2 | **-1.2** | 98.8 | 100.0 | **1.2** |
| *Physaria arenosa* | 7 | NA | NA | NA | 83.3 | 85.7 | **2.4** |
| *Picea glauca* | 7 | 44.0 | 28.6 | **-15.4** | 53.9 | 42.9 | **-11.0** |
| *Piptatheropsis pungens* | 1 | NA | NA | NA | 66.7 | 100.0 | **33.3** |
| *Plantago major* | 3 | 100.0 | 100.0 | 0.0 | 92.9 | 100.0 | **7.1** |
| *Poa pratensis* | 2 | 50.0 | 0.0 | **-50.0** | 0.0 | 0.0 | 0.0 |
| *Populus balsamifera* | 1 | 100.0 | 100.0 | 0.0 | 75.0 | 100.0 | **25.0** |
| *Populus tremuloides* | 16 | 96.4 | 100.0 | **3.6** | 89.3 | 100.0 | **10.7** |
| *Prosartes trachycarpa* | 8 | 96.3 | 87.5 | **-8.8** | 100.0 | 100.0 | 0.0 |
| *Prunus pensylvanica* | 6 | 72.7 | 83.3 | **10.6** | 52.2 | 83.3 | **31.2** |
| *Prunus virginiana* | 13 | 86.0 | 76.9 | **-9.1** | 52.9 | 61.5 | **8.6** |
| *Pulsatilla nuttalliana/Pulsatilla patens* | 9 | 86.1 | 100.0 | **14.0** | 74.4 | 88.9 | **14.5** |
| *Pyrola asarifolia* | 22 | 90.0 | 95.5 | **5.5** | 83.3 | 95.5 | **12.1** |
| *Ranunculus abortivus* | 2 | 100.0 | 100.0 | 0.0 | 45.5 | 50.0 | **4.6** |
| *Ribes hirtellum/Ribes inerme* | 3 | 6.3 | 0.0 | **-6.3** | 0.0 | 0.0 | 0.0 |
| *Ribes triste* | 2 | 100.0 | 100.0 | 0.0 | 100.0 | 100.0 | 0.0 |
| *Rosa acicularis* | 21 | 82.9 | 95.2 | **12.4** | 70.5 | 95.2 | **24.8** |
| *Rosa arkansana* | 21 | 71.2 | 76.2 | **5.0** | 67.6 | 76.2 | **8.6** |
| *Rosa woodsii* | 5 | 14.8 | 20.0 | **5.2** | 33.3 | 20.0 | **-13.3** |
| *Rubus idaeus* | 20 | 86.2 | 85.0 | **-1.2** | 64.4 | 80.0 | **15.6** |
| *Rubus pubescens* | 30 | NA | NA | NA | 89.2 | 96.7 | **7.4** |
| *Sanicula marilandica* | 6 | 100.0 | 100.0 | 0.0 | 100.0 | 100.0 | 0.0 |
| *Selaginella densa* | 30 | 100.0 | 100.0 | 0.0 | 100.0 | 20.0 | **-80.0** |
| *Solidago decemflora* | 1 | 0.0 | 0.0 | 0.0 | 0.0 | 0.0 | 0.0 |
| *Solidago gigantea* | 16 | 58.3 | 50.0 | **-8.3** | 46.8 | 56.3 | **9.5** |
| *Sphaeralcea coccinea* | 17 | 97.1 | 100.0 | **2.9** | 81.4 | 94.1 | **12.7** |
| *Sporobolus rigidus* | 20 | 55.9 | 70.0 | **14.1** | 80.5 | 90.0 | **9.5** |
| *Stachys pilosa* | 7 | 0.0 | 0.0 | 0.0 | 65.6 | 100.0 | **34.4** |
| *Stellaria longifolia* | 2 | 66.7 | 50.0 | **-16.7** | 83.3 | 100.0 | **16.7** |
| *Symphoricarpos albus* | 16 | 98.6 | 100.0 | **1.4** | 46.5 | 75.0 | **28.5** |
| *Symphyotrichum ciliolatum* | 15 | 70.7 | 73.3 | **2.6** | 67.8 | 80.0 | **12.2** |
| *Trifolium pratense* | 1 | 0.0 | 0.0 | 0.0 | 0.0 | 0.0 | 0.0 |
| *Vaccinium myrtilloides* | 15 | 98.4 | 100.0 | **1.6** | 92.1 | 100.0 | **7.9** |
| *Viburnum edule* | 12 | 100.0 | 100.0 | 0.0 | 94.6 | 100.0 | **5.4** |
| *Viburnum opulus* | 8 | 94.4 | 100.0 | **5.6** | 80.6 | 100.0 | **19.4** |
| *Vicia americana* | 9 | 76.9 | 77.8 | **0.9** | 64.1 | 44.4 | **-19.7** |
| *Viola adunca* | 2 | 63.6 | 100.0 | **36.4** | 18.2 | 50.0 | **31.8** |
| *Viola canadensis* | 6 | 95.7 | 100.0 | **4.3** | 82.6 | 100.0 | **17.4** |
| *Viola renifolia* | 9 | 77.4 | 77.8 | **0.4** | 83.9 | 100.0 | **16.1** |
| *Xanthisma spinulosum* | 10 | 86.8 | 80.0 | **-6.8** | 69.2 | 60.0 | **-9.2** |
| Summary |  |  |  |  |  |  |  |
| Minimum | 1 | 0.0 | 0.0 | -50.0 | 0.0 | 0.0 | -80.0 |
| Maximum | 48 | 100.0 | 100.0 | 36.4 | 100.0 | 100.0 | 66.7 |
| Average | 10 | 74.6 | 76.5 | 1.9 | 64.4 | 71.7 | 7.3 |

References:

Bilyk, Z. I., Shapovalov, Y. B., Shapovalov, V. B., Megalinska, A. P., Andruszkiewicz, F., & Dołhańczuk-Śródka, A. (2020). Assessment of mobile phone applications feasibility on plant recognition: comparison with Google Lens AR-app. *CEUR Workshop Proceedings*. <https://elibrary.kdpu.edu.ua/bitstream/123456789/4403/1/paper02.pdf>

Federal Geographic Data Committee. (2008). National Vegetation Classification Standard, Version 2. FGDC-STD-005-2008. Vegetation Subcommittee, Federal Geographic Data Committee, FGDC Secretariat, U.S. Geological Survey. Reston, VA

Lüdemann, J. (2020). The usability of plant identification apps and their effect on the user's engagement with species (Master's thesis). Leuphana University of Lünebrug.

Otter, J., Mayer, S., & Tomaszewski, C. A. (2021). Swipe right: a comparison of accuracy of plant identification apps for toxic plants. *Journal of Medical Toxicology*, *17*, 42-47. <https://doi.org/10.1007/s13181-020-00803-6>

Schmidt, M., & Steinecke, H. (2019). Heimische Pflanzen mit dem Smartphone bestimmen–ein Praxistest. Der Palmengarten, *83*(2), 138-140.
